# Microbial metabolic potential of hydrothermal vent chimneys along the submarine ring of fire

**DOI:** 10.3389/fmicb.2024.1399422

**Published:** 2024-08-06

**Authors:** Laura Murray, Heather Fullerton, Craig L. Moyer

**Affiliations:** ^1^Department of Biology, Western Washington University, Bellingham, WA, United States; ^2^Department of Biology, College of Charleston, Charleston, SC, United States

**Keywords:** hydrothermal vents, chimneys, community structure, metagenomics, metabolism

## Abstract

Hydrothermal vents host a diverse community of microorganisms that utilize chemical gradients from the venting fluid for their metabolisms. The venting fluid can solidify to form chimney structures that these microbes adhere to and colonize. These chimney structures are found throughout many different locations in the world’s oceans. In this study, comparative metagenomic analyses of microbial communities on five chimney structures from around the Pacific Ocean were elucidated focusing on the core taxa and genes that are characteristic of each of these hydrothermal vent chimneys. The differences among the taxa and genes found at each chimney due to parameters such as physical characteristics, chemistry, and activity of the vents were highlighted. DNA from the chimneys was sequenced, assembled into contigs, and annotated for gene function. Genes used for carbon, oxygen, sulfur, nitrogen, iron, and arsenic metabolisms were found at varying abundances at each of the chimneys, largely from either Gammaproteobacteria or Campylobacteria. Many taxa shared an overlap of these functional metabolic genes, indicating that functional redundancy is critical for life at these hydrothermal vents. A high relative abundance of oxygen metabolism genes coupled with a low abundance of carbon fixation genes could be used as a unique identifier for inactive chimneys. Genes used for DNA repair, chemotaxis, and transposases were found at high abundances at each of these hydrothermal chimneys allowing for enhanced adaptations to the ever-changing chemical and physical conditions encountered.

## 1 Introduction

Deep-sea hydrothermal vents have been an ecosystem of interest since their discovery in 1977 (Francheteau et al., 1979). The mixing of the hot reduced fluids with the cold oxygenated seawater creates the chimney, a solid structure to which organisms can adhere (Opatkiewicz et al., 2009). Not only do these chimneys provide a surface for microbial growth but also vent fluids support the growth of chemoautotrophic microbes (Kato et al., 2018). The metabolic byproducts of these microbes are disseminated throughout the ocean, contributing to global geochemical cycling functioning as the trophic base to both deep-sea and upper ocean food webs (Edwards, 2004; Ardyna et al., 2019). By metabolizing vent fluid and mineral deposits of the hydrothermal vent chimneys, chemoautotrophic microbes enhance community richness and diversity by allowing for hydrothermal vents to become more diverse communities (Wright et al., 2002).

Hydrothermal vents are ecologically and economically important. A sizeable fraction of the ocean’s organic carbon is a direct result of microbial carbon fixation from these venting sites (Cathalot et al., 2021). Similarly, microbial production of iron at hydrothermal vents is critical for primary productivity and nitrogen fixation throughout the deep ocean (Früh-Green et al., 2022). Due to the high levels of valuable metals such as silver, copper, cobalt, and gold, hydrothermal vent systems are increasingly being considered for deep-sea mining (Han et al., 2018). Deep-sea mining produces large particle plumes and landslides that can impact benthic filter feeders’ ability to access food and destroy the growth surfaces (Orcutt et al., 2020). This could have downstream impacts since these microbes could act as settlement cues for vent-endemic invertebrates (O’Brien et al., 2015). Without these microbial primary producers at these chimney sites, the rich ecosystem would become largely uninhabitable.

Chimneys are found worldwide and have a wide range of venting temperatures, chemical composition, and flow rates (Emerson and Moyer, 2010). Differences in abiotic factors, such as the presence of oxygen, pH, pressure, heat flux, and reduced chemicals, dictate the biogeographical distribution of microbes. These differences allow for microbes to partition specific niches at venting sites, dictated by the availability of compounds such as sulfide and carbon in the venting fluid and sediment (Meier et al., 2017). However, due to the necessity of microbes having optimal conditions needed for growth, vents with similar environmental characteristics have similar microbial communities (Dick, 2019).

**Figure 1:**
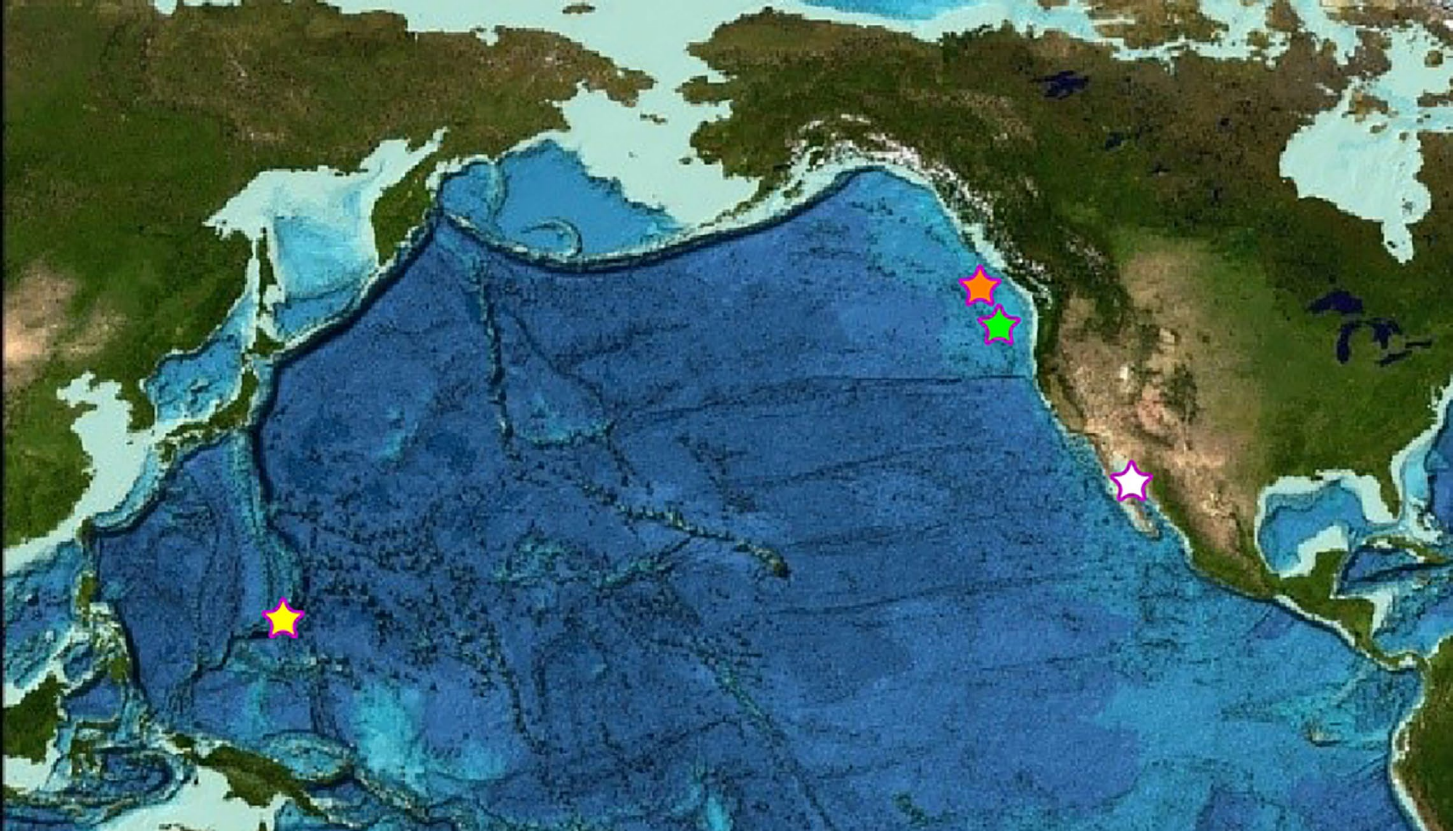
Map of the four sampling locations across the Pacific Ocean. The orange star denotes the Magic Mountain, Explorer Ridge sampling location of the Ochre Chimney, and the green star denotes the Axial Volcano, Juan de Fuca Ridge sampling location of the Castle Chimney. The white star denotes the Guaymas Basin sampling location of the Pagoda Chimney. The yellow star denotes the Mariana back-arc Urashima sampling location of the Snap-Snap and Ultra-No-Chi-Chi Chimneys (Image reproduced from the GEBCO world map 2019, www.gebco.net).

**Figure 2:**
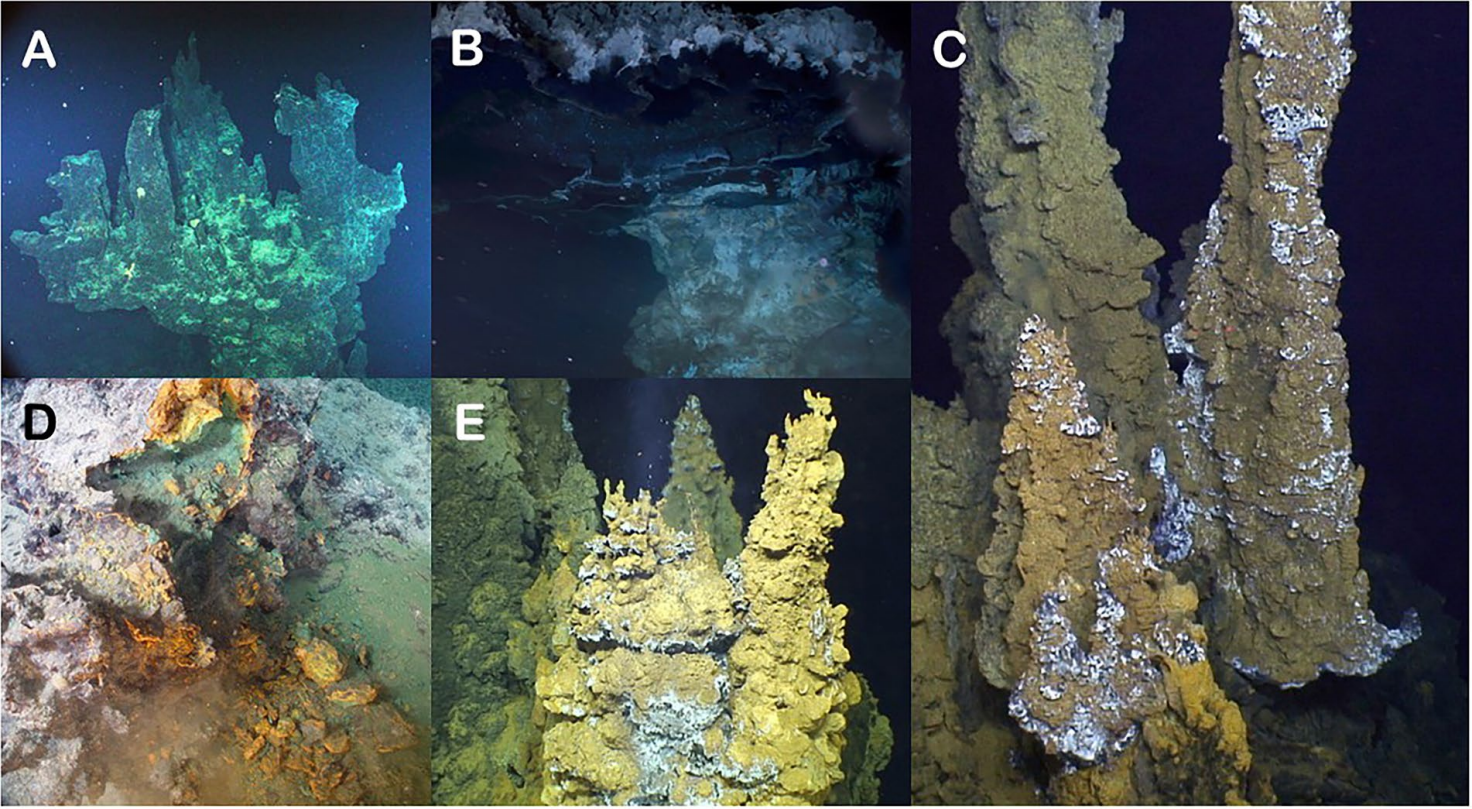
Photographs of the five chimneys evaluated in this study. **(A)** Castle Chimney from Axial Seamount. **(B)** Pagoda Chimney from Guaymas Basin. **(C)** Ultra-No-Chi-Chi Chimney from the Urashima Vent Field (Laser dots are 10 cm apart). **(D)** Ochre Chimney from Magic Mountain. **(E)** Snap-Snap Chimney also from the Urashima Vent Field.

In this study, we sought to characterize the microbial community members and metabolic potential of five different chimneys at four locations found along the plate boundary in the Pacific Ocean, known as the “Submarine Ring of Fire” (Figure 1). This approach allowed for an examination of the similarities and differences in microbial community structure and identification of the common genes found at each chimney across these selected vent chimneys (Figure 2).

Axial Seamount (Juan de Fuca Ridge) is an active submarine volcano and a site of extensive hydrothermal venting, with the most recent eruption occurring in 2015 (Chadwick et al., 2016). The high volcanic activity of this caldera produces a large amount of hydrogen sulfide, ferrous oxide, and methane that are released during eruption events. An abundance of sulfur-oxidizing bacteria and methanogenic archaea correlate with high concentrations of hydrogen sulfide and methane (Topçuoğlu et al., 2016). The geochemistry of the venting sites is important in shaping community structure and genes present at each vent (Fortunato et al., 2018). For this site, we investigated the Castle Chimney, located on the eastern side of Axial Caldera (Figure 2A).

The Guaymas Basin (Gulf of California) hydrothermal vents are highly active with steep temperature gradients and covered with a layer of organic-rich sediments. Some chimneys here are characterized not by direct venting but by internal hydrothermal fluid circulation due to their shape (Teske et al., 2016). These unique chimneys have pagoda-like structures that have fluid circulating internally, leading to large, temperature gradients (Figure 2B). Metagenomic analysis of Guaymas Basin sediments showed an enrichment of genes for methane, hydrogen, and sulfide metabolisms as compared to background sediments (Dombrowski et al., 2018).

At the Urashima Vent Field (Mariana back-arc), dissolved sulfide and hydrogen concentrations in vent effluent are enhanced. Due to the high levels of sulfide, the microbial community is dominated by the sulfur-oxidizing Campylobacteria (Trembath-Reichert et al., 2019). These were formerly known as Epsilonproteobacteria (Waite et al., 2017). Urashima Vents have been shown to have a relatively high abundance of Zetaproteobacteria, a class of iron-oxidizing bacteria that forms dense mat structures made from iron oxides and polysaccharides which are used as a colonizing surface for other microbial taxa (McAllister et al., 2020). At this location, we investigated both the Ultra-No-Chi-Chi and Snap-Snap Chimneys (Figures 2C,E).

Magic Mountain (Explorer Ridge) has over 50 active and inactive vents, including the inactive Ochre Chimney (Figure 2D), which still maintained diffuse fluid flow based on the associated Fe and H_2_S geochemistry (Fullerton et al., 2024). The active and inactive chimney structures found in this vent field are composed largely of sulfide deposits (Fortin et al., 1998). When examining inactive chimneys in other locations, sulfide-oxidizing Gammaproteobacteria tend to dominate the microbial communities as they can use metal sulfide present in the chimney structures and mineral deposits for energy (Edwards et al., 2003; Sylvan et al., 2012; Meier et al., 2019; Achberger et al., 2024).

The metabolic byproducts of microorganisms that form and reside in hydrothermal vent chimneys facilitate nutrient cycling in both the hydrothermal vent ecosystem and throughout the world’s oceans. Diverse communities of microbes utilize chemicals in the venting fluid to gain energy and biomass. Here, metagenomic and amplicon sequencing was used to identify metabolism genes to better understand the metabolic potential of chimneys. The combination of genes detected in this study sheds light on hydrothermal vent chimneys’ community structure and metabolic potential throughout the Pacific Ocean.

## 2 Methods

### 2.1 Study sites and sampling

Chimneys were collected from Axial Seamount, Magic Mountain, Guaymas Basin, and two chimneys from the Urashima Vent Field along the Mariana back-arc using either a self-sealing scoop sampler or biobox. At Axial Seamount, the Castle Chimney was collected on 4 August 2002 with ROV ROPOS aboard the R/V Thomas G. Thompson. The Ochre Chimney at Magic Mountain Vent Field was collected on 29 July 2002 with ROV ROPOS aboard the R/V Thomas G. Thompson. The Pagoda Chimney at the Guaymas Basin Vent Field was collected on 7 October 1994 with HOV ALVIN aboard the R/V Atlantis. Two chimney samples, namely, Snap-Snap and Ultra-No-Chi-Chi, were collected with ROV Jason aboard the R/V Roger Revelle from the Urashima Vent Field. The Snap-Snap Chimney was sampled on 1 November 2014, and the Ultra-No-Chi-Chi Chimney was sampled on 18 December 2014 (Table 1). All chimney samples from all sites were immediately preserved with RNAlater and then stored at −80°C until DNA could be extracted. Geochemistry data from nearby chimneys or their venting fluid were sourced from previous publications for the Pagoda Chimney (Von Damm et al., 1985), the Castle Chimney (Butterfield et al., 1990; Fortunato et al., 2018), the Ochre Chimney (Fullerton et al., 2024), and the two Urashima Chimneys (Hager et al., 2017; Table 1).

**Table 1:** Summary of samples collected from five different hydrothermal vent chimneys.

Chimney sample	Dive number	Collection DSV	Date	Latitude and longitude	Depth (m)	Temp (°C)	Fe (μM)	H_2_S (μM)	H_2_ (μM)
Magic Mountain: Ochre	R668	ROV ROPOS	07/29/2002	49°45.38’N, 130°15.75’W	1847	4	12 to 20^A^	315 to 559^A^	nd
Axial: Castle	R674	ROV ROPOS	08/04/2002	45°55.57’N, 129°58.80’W	1,522	235	31^B^	7100^B^	1.5^B^
Guaymas: Pagoda	A2838	HOV ALVIN	10/07/1994	27°00.91’N, 111°24.64’W	1980	279	17 to 180^C^	3,800 to 5980^C^	nd
Urashima: Snap-Snap	J2-797	ROV JASON II	11/01/2014	12°55.33’N, 143°38.95’W	2,928	161	48.5^D^	<0.4^D^	0.01^D^
Urashima: Ultra-No- Chi-Chi	J2-801	ROV JASON II	12/18/2014	12°55.34’N, 143°38.95’W	2,929	174	48.5^D^	<0.4^D^	0.01^D^

^A^Chemistry data for the Ochre Chimney were taken from Fullerton et al. (2024). ^B^Fe and H_2_S data for the Castle Chimney were taken from the venting fluid of a nearby chimney from Butterfield et al. (1990). H_2_ data were taken from the venting fluid from Fortunato et al. (2018). ^C^Chemistry data for the Pagoda Chimney were taken from Von Damm et al. (1985). ^D^Chemistry data represent venting fluid from the Snap-Snap and Ultra-No-Chi-Chi Chimneys and were taken from Hager et al. (2017).

### 2.2 Amplicon DNA extractions, sequencing, and sequence processing

Genomic DNA was extracted from cell pellets using the Fast DNA SPIN Kit for Soil (MP Biomedicals, Santa Ana, CA) as previously published (Hager et al., 2017). Cell lysis was optimized using two rounds of bead beating for 45 s at a power setting of 5.5 using the FastPrep instrument (MP Biomedicals) with samples being placed on ice between runs. Extracted DNA was quantified by a Qubit 2.0 fluorometer using high-sensitivity reagents (Thermo Fisher Scientific, Waltham, MA).

The V3-V4 regions of the SSU rRNA gene were amplified via PCR-purified DNA using bacterial primers 340F and 784R (Klindworth et al., 2013; Hager et al., 2017). The resulting amplicons were sequenced using a MiSeq (Illumina, San Diego, CA) as per the manufacturer’s protocol generating 2 × 300 bp paired-end reads. The resulting reads were trimmed out of primers using Cutadapt (Martin, 2011). The trimmed reads were then processed using the Divisive Amplicon Denoising Algorithm 2 (DADA2) v1.26.0 with pseudopooling following the previously described protocols (Lee et al., 2015; Callahan et al., 2016, 2017) with R version 4.2.3 and using the Silva v138 database for assigning taxonomy. Further analysis was completed using phyloseq version 1.32 (McMurdie and Holmes, 2013) and microbiome version 1.20 (Lahti and Shetty, 2017).

### 2.3 Metagenomic sequencing

The extracted DNA was separated into strands greater than 1 kb using an Aurora (Boreal Genomics, Vancouver, BC). Nextera indices were added to purified DNA fragments as per the manufacturer’s protocol (Illumina, San Diego, CA). The indexed fragments were purified using AMPure XP Beads according to the manufacturer’s protocol (Beckman Coulter, IN). The library was quantified with a Qubit 2.0 fluorometer (Thermo Fisher, MA) and sequenced on the Illumina MiSeq sequencer with v3.0 chemistry to generate 2×300 bp paired-end reads at Shannon Point Marine Center, WWU.

### 2.4 Metagenomic sequence analysis

After sequencing, the reads were assessed and trimmed for quality control using the program Trimmomatic v.0.40 (Bolger et al., 2014) and were assessed for quality with FastQC v.0.11.9 (Andrews, 2010). All metagenomic analyses were done under the metagenomic pipeline, SqueezeMeta v.1.5.1 (Tamames and Puente-Sánchez, 2019), under co-assembly mode using default parameters. Once trimmed for quality, the reads were assembled into contigs using MEGAHIT with a minimum contig length of 200 base pairs (Li D. et al., 2015). The assembled contigs were checked for rough taxonomy using MetaQuast (Mikheenko et al., 2016). Genes were predicted using Prodigal (Hyatt et al., 2010). Small subunit (SSU) rRNA genes were pulled from contigs using Barrnap with a minimum length requirement of 200 base pairs (Seemann, 2014) and classified using the RDP naïve Bayesian classifier (Wang et al., 2007).

Gene sequences were compared for homology using Diamond v.2.7.14 (Buchfink et al., 2015). The genes were then functionally and taxonomically assigned using SqueezeMeta by running a Diamond search against the COG and KEGG databases for functional assignments and GenBank nr for taxonomic assignments. Either a best hit or best average method is used for assigning functional IDs to each hit, or the average of 5 best hits for any given functional ID. For taxonomic assignment, an LCA of the best hits is obtained, where the hits had to pass a minimum amino acid identification level to be used for assigning taxonomic ranks. These thresholds are 85, 60, 55, 50, 46, 42, and 40% for species, genus, family, order, class, phylum, and superkingdom ranks, respectively. Bowtie2 v.2.4.5 was used to estimate coverage and abundance (Langmead and Salzberg, 2012). Gene abundances were calculated using STAMP (Parks et al., 2014), and then the percent relative abundance of each gene was calculated by taking the raw read counts for each ORF, dividing it by the total reads in the sample, and multiplying by 100. Coverage values (bases mapped/ORF length) and normalized RPKM values were calculated using custom SqueezeMeta pipeline scripts. All outputs of taxonomic names present in this analysis reflect the names in the databases at the time of assembly and assignment. Contig assemblies were loaded into FeGenie to evaluate the abundance of different iron genes in each chimney (Garber et al., 2020). Outputs were visualized in R version 4.2.3 with the package ggplot2 v.3.3.5 (Wickham, 2016).

After assembly, the contigs were binned into metagenomic assembled genomes (MAGs) using Metabat2 v.2.15 (Kang et al., 2015) and Maxbin2 v.2.2.7 (Wu et al., 2016). The outputs of both binning programs were merged using DAStool v.1.1.3 (Sieber et al., 2018). Bins were checked for completeness and contamination using the program CheckM v.1.1.3 (Parks et al., 2015) and then taxonomically assigned using a Diamond homology search with an LCA algorithm in SqueezeMeta.

In R v.3.6.3, the results of the SqueezeMeta co-assembly pipeline were imported using the R package SQMtools v.0.7.0 (Puente-Sánchez et al., 2020). The Vegan package (Oksanen et al., 2008) was used to conduct permutational multivariate analysis of variance (PERMANOVA) among the chimneys with 999 permutations and the Bray–Curtis distance method, non-metric multidimensional scaling (NMDS) based on Bray–Curtis distance of KEGG functions, and to assess the relative abundance and diversity of the taxa found at each chimney. The similarity of taxa was analyzed using the Bray–Curtis distance matrix and visualized with a dendrogram.

## 3 Results

### 3.1 Assembly

After sequencing and filtration for quality, 207,450,568 reads were assembled into 15,601,568 contigs. The N50 was 385 base pairs which had an average coverage of 0.669 at Ochre, 0.815 at Castle, 0.515 at Pagoda, 0.388 at Snap, and 0.446 at Ultra. After composite assembly, a total of 17,978,241 ORFs were identified, and of these, 40.7% were able to be annotated. The composite assembly resulted in 43 MAGs with a completeness range of 10 to 52% and a contamination range of 0 to 4.76% (Supplementary Table S1). Due to the low completeness and high contamination level of the MAGs, further analysis was done directly on the composite assembly (Bowers et al., 2017).

### 3.2 Alpha and beta diversity

**Figure 3:**
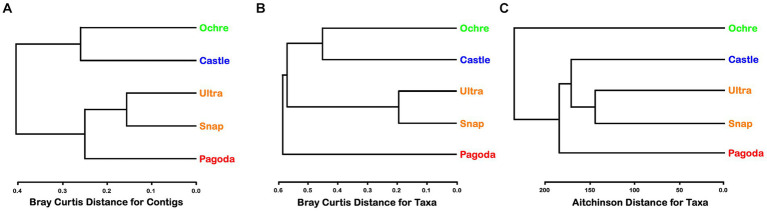
**(A)** Cluster analysis of metagenomic-derived contig similarities among chimneys using Bray–Curtis distance for contigs. Green represents the Ochre Chimney at Magic Mountain, blue represents the Castle Chimney at Axial Seamount, orange represents the Snap-Snap and Ultra-No-Chi-Chi Chimneys from the Urashima Vent Field, and red represents the Pagoda Chimney at Guaymas Basin Vent Field. **(B)** Cluster analysis of metagenomic-derived taxa similarities among chimneys using Bray–Curtis distance for taxa. **(C)** Cluster analysis of amplicon sequencing-derived taxa similarities among chimneys using Aitchison distance for taxa.

In general, alpha diversity was greater in the amplicons than in the metagenomic contigs. The Pagoda Chimney had the highest alpha diversity of both Shannon and Simpson indices in contigs, while the Ochre Chimney had the highest alpha diversity regarding amplicon-generated ASVs (Supplementary Table S2). Dendrograms for the metagenomic function, metagenomic-derived taxa identification, and SSU rRNA communities varied in branching pattern and out-groups across the Chimneys (Figure 3). Clustering of chimneys using a dendrogram of Bray–Curtis distance matrices of KEGG gene functions showed the inactive Ochre Chimney paired with the Castle Chimney, while the two Urashima Chimneys clustered together with Pagoda as an out-group (Figure 3A). When clustered by metagenomic-derived taxa, the Pagoda Chimney was the out-group, while the two Urashima chimneys clustered most closely together, and the Castle and Ochre chimneys also clustered together (Figure 3B). The amplicon sequence analysis showed the two Urashima Chimneys cluster most closely together, similar to the SSU rRNA genes from the composite assembly (Figure 3C). However, with the amplicon analysis, the Ochre Chimney was the out-group. PERMANOVA analysis was run to test the variance of the chimneys by location and metagenomic contig composition. Despite each chimney having a significant R^2^ value after PERMANOVA analysis, the *p*-value was 0.1 or greater, likely due to each chimney only having a single sample.

**Figure 4:**
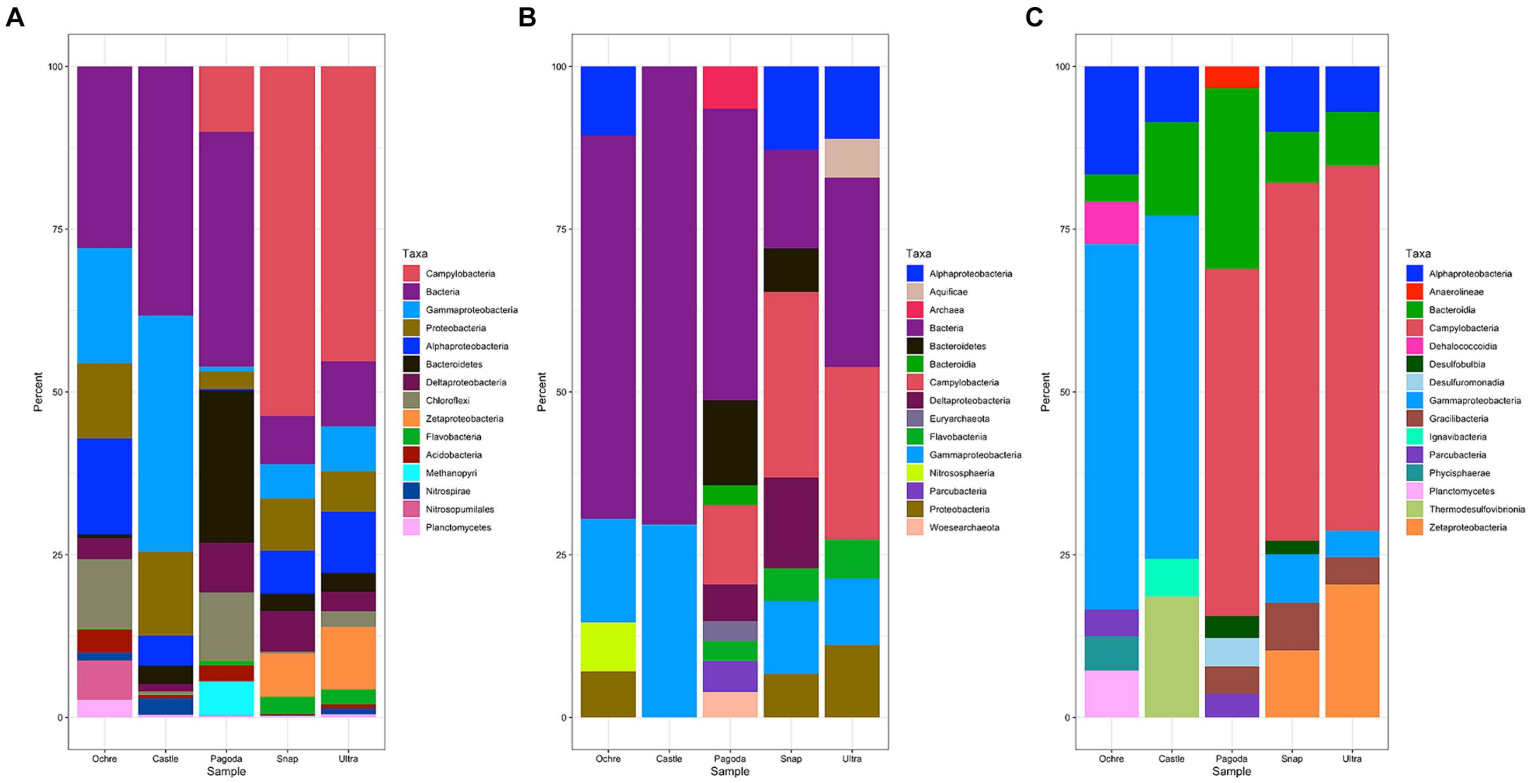
Stacked bar graph of the top 15 microbial taxa found in each chimney as a percentage of reads from the whole metagenome and from all taxa detected by amplicon sequencing. **(A)** Stacked bar graph of the ORFs from assembly, not including unclassified or unmapped reads. **(B)** Stacked bar graph of the SSU rRNA gene reads, only including the top 15 taxa, not including unclassified or unmapped metagenome reads. **(C)** Stacked bar graph based on amplicon sequencing of SSU rRNA genes using V3V4 primers.

Each chimney had variations in the taxonomic identity of the ORFs, the identity of SSU rRNA genes in the composite assembly, and SSU rRNA amplicon identity (Figure 4). The composite metagenomes resulted in the identification of 23,040 SSU rRNA genes, whereas amplicon analysis resulted in 5,380 ASVs. By ORF analysis, Deltaproteobacteria were identified in all five chimneys (Figure 4A). However, by metagenomic SSU rRNA genes, Deltaproteobacteria were identified in Pagoda and Snap-Snap, and by amplicon sequencing, Deltaproteobacteria were identified only in the Castle Chimney (Figures 4B,C). Analysis of ORFs differed from that of metagenomic SSU rRNA genes and amplicon sequencing in that Alphaproteobacteria were found present in the Pagoda Chimney (Figure 4A). Chloroflexi were found present in the Ochre and Pagoda Chimneys by ORF analysis but were absent in the other two analyses. ORF analysis also identified Nitrospirae and Nitrosopumilales in the Ochre Chimney, while neither of these taxa were present in metagenomic SSU rRNA genes or amplicon sequencing (Figure 4).

When evaluating metagenomic SSU rRNA genes, more Archaeal taxa were identified in the Pagoda Chimney than in the other two methods of identification. Much larger proportions of the taxa were only assigned to domain level, indicating that this method of taxonomic identification is likely less refined than the other two methods (Figure 4B). Bacteroidetes were only found to be present in the Pagoda Chimney and Snap-Snap Chimney when examining metagenomic SSU rRNA genes but were found in all five chimneys when looking at ORFs (Figures 4A,B).

Amplicon sequencing of the SSU rRNA gene demonstrated a lack of Flavobacteriia that were otherwise found in the Pagoda, Snap-Snap, and Ultra-No-Chi-Chi Chimneys from ORF analysis and in the Pagoda and Snap-Snap Chimneys from metagenomic SSU rRNA gene analysis (Figure 4). Bacteroidia were found at all five chimneys using amplicon sequencing, while they were only found in the Pagoda Chimney when examining metagenomic SSU rRNA genes and were absent from all five chimneys using ORF analysis. Amplicon sequencing also identified much larger proportions of Campylobacteria in the Pagoda Chimney than the other two methods of analysis (Figure 4C). A few taxa were identified from amplicon sequencing that were not found in the other two methods of analysis, including Anaerolineae, Dehalococcoidia, Desulfobulbia, Desulfuromonadia, Gracilibacteria, Ignavibacteria, Phycisphaerae, and Thermodesulfovibrionia. Parcubacteria were found present in both the Ochre Chimney and the Pagoda Chimney using amplicon sequencing, were found present in the Pagoda Chimney from only metagenomic SSU rRNA genes, and were not found using ORF analysis (Figure 4).

### 3.3 Gene abundances

**Figure 5:**
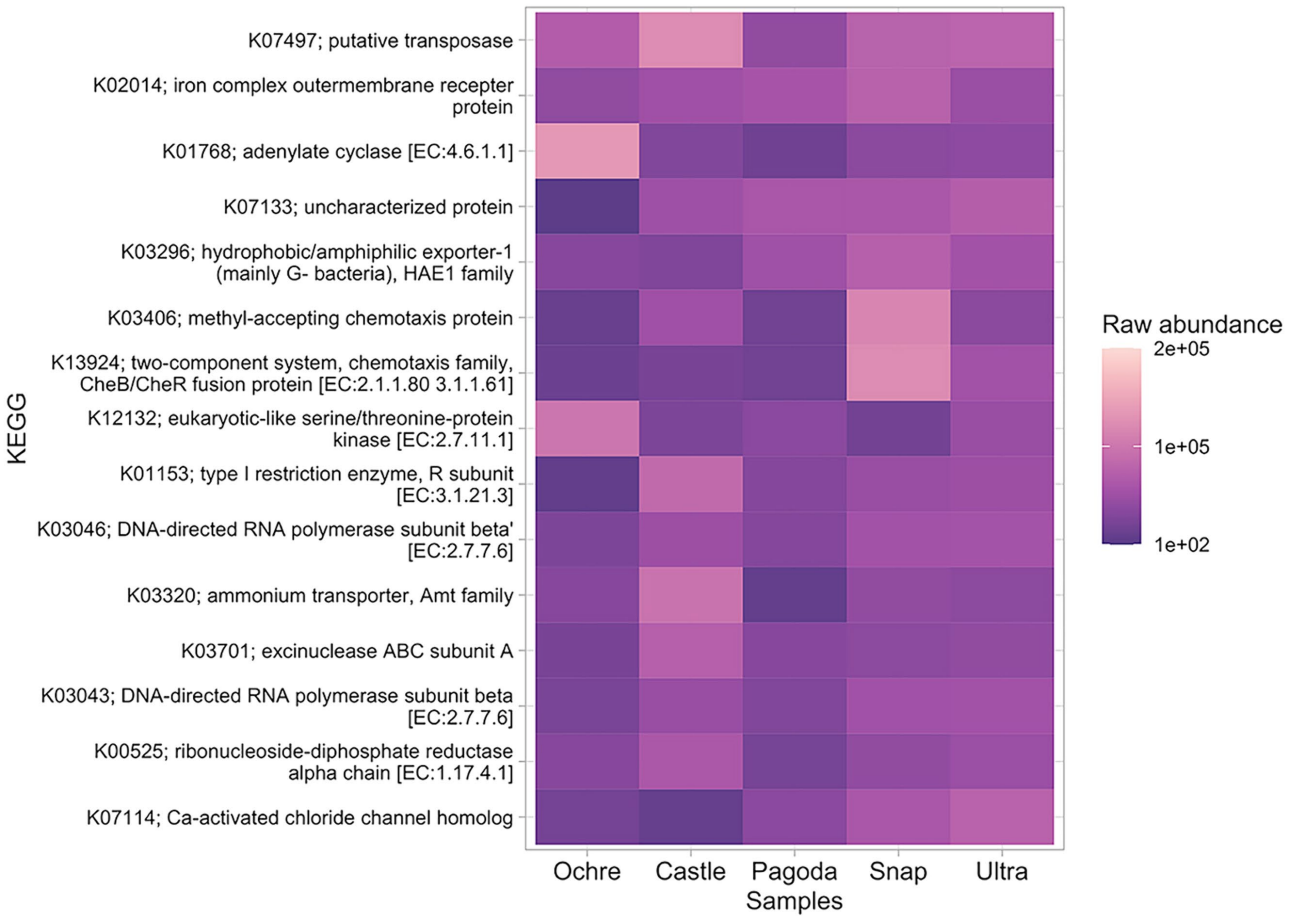
Heatmap of the top 15 most abundant KEGG genes found in all five chimneys. Presence is measured in raw abundance of reads.

The most abundant gene present in all chimneys was a putative transposase gene (Figure 5). Notably, an ammonium transporter gene was also present in the top 15 most abundant genes, with higher abundance at the Castle Chimney and lower abundance at the Pagoda Chimney. DNA-directed RNA polymerase was present at all chimneys, with higher abundances in Ochre and Pagoda. Two chemotaxis genes were found at high abundance in the Snap-Snap Chimney but less abundant in the other chimneys. Genes for motility, signaling and cellular processes, and folding and degradation were found to be largely differentially abundant among chimneys (Supplementary Figure S1A). When comparing differentially abundant genes between the active chimney and inactive chimney, hydrogenases were found to be more differentially abundant in the active chimneys while dehydrogenases were more differentially abundant in the Ochre Chimney (Supplementary Figure S1B).

### 3.4 Metabolic potential

#### 3.4.1 Carbon cycling

**Figure 6:**
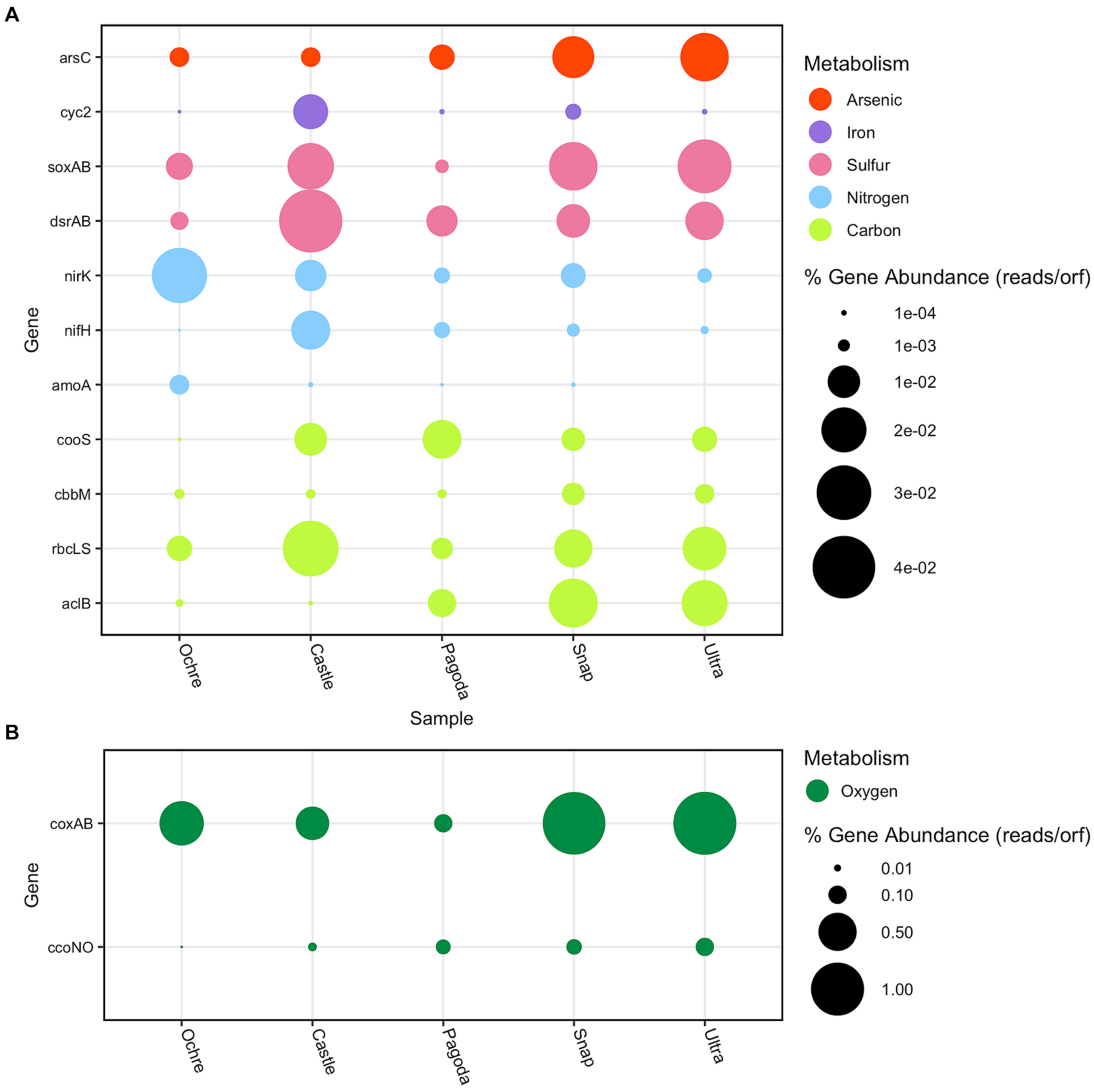
Bubble plot of the relative abundance of key metabolic genes found in each chimney. Relative abundance is measured in the number of reads per ORF divided by the total number of reads. **(A)** Arsenic, iron, sulfur, nitrogen, and carbon genes. The red bubbles represent arsenic cycling genes, the purple bubbles represent iron cycling genes, the pink bubbles represent sulfur cycling genes, the blue bubbles represent nitrogen cycling genes, and the lime green bubbles represent carbon cycling genes. **(B)** Oxygen metabolism genes are represented by dark green bubbles.

Chemosynthetic primary producers at hydrothermal vent chimneys predominately fix carbon via the reverse tricarboxylic acid cycle (rTCA), the Calvin–Benson–Bassham (CBB) cycle, and the Wood–Ljungdahl (WL) pathway (Erb, 2011; Hügler and Sievert, 2011; Mall et al., 2018). The rTCA cycle uses the enzyme ATP citrate lyase (*aclB*) to take CO_2_ and water to make carbon compounds that can be used for energy by microorganisms in low-oxygen environments (Mall et al., 2018). The *aclB* gene was found to be present in all five chimneys (Figure 6). The Castle and Ochre Chimneys both had Nitrospirae and Campylobacteria identified *aclB* genes (Supplementary Tables S3, S4). The Pagoda Chimney *aclB* was found in unclassified archaea and bacteria, as well as Thermoplasmata, Candidatus Bipolaricaulota, Chloroflexi, Aquificae, and Campylobacteria. At the Snap-Snap and Ultra-No-Chi-Chi Chimneys, Campylobacteria *aclB* genes dominated in relative abundance (Supplementary Tables S5–S7).

The CBB cycle was identified using the RuBisCO protein, shown here by the presence of type I and type II RuBisCO genes, *rbcLS*, and *cbbM* respectively, which have varying affinities for oxygen (Tabita et al., 2008). The *rbcLS* RuBisCO gene has a lower affinity for oxygen, while *cbbM* has a higher affinity (Rickaby and Eason Hubbard, 2019). These genes were found to be differentially abundant at all five chimneys, with *rbcLS* dominating. The *rbcLS* ORFs found were identified as Alphaproteobacteria, Chloroflexi, Proteobacteria, and Gammaproteobacteria across all five chimneys (Figure 6; Supplementary Tables S3–S7). The Castle and Ochre Chimneys showed similar assignments of *rbcLS*-assigned taxa, with Proteobacteria dominating and a low presence of archaeal *rbcLS* genes (Supplementary Tables S3, S4). In contrast, the Pagoda Chimney and both the Urashima Chimneys showed a higher presence of archaeal *rbcLS* genes (Supplementary Tables S5–S7). The *cbbM* gene was identified as Alphaproteobacteria in every chimney and Gammaproteobacteria in every chimney but Pagoda, which was dominated by Methanomicrobia and Thermococci. The two Urashima Chimneys had a large abundance of Zetaproteobacterial *cbbM* genes (Supplementary Tables S6, S7).

The Wood–Ljungdahl pathway reduces CO_2_ to carbon monoxide using the enzyme carbon monoxide dehydrogenase (*cooS* and *acsA*, both represented here by *cooS*) and then creates acetyl-CoA using acetyl-CoA synthase (Ragsdale and Pierce, 2008). The Wood–Ljungdahl pathway was found to be present across all five chimneys. This gene was least abundant at the Ochre Chimney and most abundant at the Pagoda Chimney (Figure 6). Deltaproteobacteria, Gammaproteobacteria, and Nitrospirae identified *cooS* all were found at higher relative abundances at the Castle Chimney compared to the other chimneys. Chloroflexi, Deltaproteobacteria, and Methanopyri *cooS* were the most abundant at the Pagoda Chimney, and Deltaproteobacteria and Nitrospirae *cooS* were found to be in the highest relative abundance at the two Urashima Chimneys (Supplementary Tables S3–S7).

#### 3.4.2 Nitrogen cycling

Three nitrogen cycling genes were annotated and taxonomically assigned for each chimney: methane/ammonia monooxygenase subunit A (*amoA*), nitrogenase iron protein (*nifH*), and nitrite reductase (*nirK*), a gene responsible for a step in the denitrification pathway, facilitating the reduction of nitrite to nitric oxide (NO). The least abundant nitrogen cycling gene detected across all five chimneys was *amoA* (Supplementary Tables S3–S7). Nitrification as represented by the *amoA* gene showed the greatest representation in the Ochre Chimney, with corresponding taxa including Betaproteobacteria, Deltaproteobacteria, Gammaproteobacteria, Nitrososphaeria, unclassified Thaumarchaeota, and unclassified Archaea (Supplementary Table S3). Interestingly, *amoA* is completely absent from the Ultra-No-Chi-Chi Chimney, while this gene was identified at Snap-Snap, its geographic neighbor.

The Pagoda Chimney had the lowest total abundance of nitrogen metabolism genes compared to the other chimneys. Dinitrogen fixation by *nifH* was dominated by Methanopyri, Nitrospirae, Firmicutes, Gammaproteobacteria, and Deltaproteobacteria (Figure 6, Supplementary Table S5). Most of the ORFs assigned to *nifH* genes were found to be associated with Methanopyri, a class of methanogenic Euryarchaeota that can be nitrogen fixers (Leigh, 2000; Auman et al., 2001; Bae et al., 2018). The Castle Chimney had a large proportion of *nifH* genes dominated by Proteobacteria, while the two Urashima Chimneys had small abundances of *nifH* genes identified as Deltaproteobacteria, Thermodesulfobacteria, and Archaeoglobi compared to the other chimneys (Supplementary Tables S3, S6, S7).

The most prevalent form of potential nitrogen metabolism across the samples except the Pagoda and Castle Chimneys was denitrification via *nirK* (Figure 6). Each chimney had a unique taxonomic distribution of *nirK* taxa, with high relative abundances in Bacteroidetes, Gammaproteobacteria, and unclassified Bacteria. The Ochre Chimney had the highest diversity of *nirK* genes across both archaea and bacteria, with most of the reads assigned to Nitrososphaeria (Supplementary Tables S3–S7).

#### 3.4.3 Sulfur cycling

Two sulfur cycling genes were examined in all five chimneys: dissimilatory sulfite reductase *dsrAB* and thiosulfotransferase *soxAB*. Across all five chimneys, *dsrAB* was found present in Alphaproteobacteria, Deltaproteobacteria, Gammaproteobacteria, Proteobacteria, and unclassified Bacteria. It was also found in the four active chimneys as Acidobacteria and Nitrospirae. The *soxAB* genes were represented across similar taxa as *dsrAB* genes in all five chimneys. At the Ochre Chimney, the Gammaproteobacterial and Alphaproteobacterial *dsrAB* genes found are the oxidative version of *dsrAB*, indicating that these organisms are likely sulfur-oxidizing bacteria. Alphaproteobacteria, Gammaproteobacteria, and unclassified Bacteria *dsrAB* genes were present in all chimneys, and Deltaproteobacteria and Campylobacteria were present in the four active chimneys (Supplementary Tables S3–S7).

#### 3.4.4 Iron cycling

To observe iron metabolisms in the chimneys, the iron oxidase gene *cyc2* was examined since it is useful as an indicator of microbial iron oxidation (McAllister et al., 2020). The presence of iron was confirmed previously at all chimneys (Table 1). However, *cyc2* genes were found present in both the Pagoda Chimney and the Ochre Chimney. The most abundant *cyc2* gene taxon was Gammaproteobacteria, found in all chimneys except for Ultra-No-Chi-Chi (Supplementary Tables S3–S7). At the Snap-Snap Chimney, Alphaproteobacteria, Gammaproteobacteria, Bacteroidetes, and unclassified Bacteria had the *cyc2* gene. In contrast, Ultra-No-Chi-Chi only had ORFs assigned to Proteobacteria and Aquificae *cyc2* genes (Supplementary Tables S3–S7). In FeGenie, *cyc2* was not identified (Supplementary Figure S2). The iron complex outer membrane receptor gene, involved in the acquisition and uptake of iron, was found to be present across all five chimneys, with the largest relative abundance found in the Snap-Snap Chimney (Figure 5).

#### 3.4.5 Arsenic metabolism

Arsenate reductase, *arsC*, allows for the reduction of arsenate, and each of the five chimneys contained this gene in at least 12 different taxa including Gammaproteobacteria, Bacteroidetes, Proteobacteria, and unclassified Bacteria (Supplementary Tables S3–S7). The Ultra-No-Chi-Chi Chimney had the most *arsC* genes present. In the Castle and Ochre Chimneys, Gammaproteobacteria had the most ORFs assigned, while Campylobacteria dominated the two Urashima Chimneys. The Pagoda Chimney had the largest abundance of Bacteroidetes identified *arsC* genes (Supplementary Tables S3–S7).

#### 3.4.6 Oxygen metabolism

The relative abundance of two cytochrome c oxidase genes (*ccoNO* and *coxAB*) was evaluated as an indicator of aerobic respiration, and in general, *coxAB* was more abundant across the five chimneys. The *ccoNO* gene was more abundant at the Ultra-No-Chi-Chi, Snap-Spap, and Pagoda Chimneys, with the least amount detected at the Castle and Ochre Chimneys (Figure 6). At the Ochre Chimney, Bacteroidetes, Flavobacteriia, and Gemmatimonadetes had the highest relative abundance of ORFs assigned to *ccoNO* genes (Supplementary Table S2). The Castle Chimney had a large abundance of Bacteroidetes and Gammaproteobacteria *ccoNO* genes (Supplementary Table S4). Chlorobi, Flavobacteriia, Bacteroidetes, and Cytophagia *ccoNO* genes were most abundant at the Pagoda Chimney (Supplementary Table S4). At Snap-Snap; Acidobacteria, Bacteroidetes, Chlorobi, and Flavobacteriia had the most *ccoNO* genes assigned (Supplementary Table S6). Ultra-No-Chi-Chi had a similar abundance of *ccoNO* genes, with the addition of Deltaproteobacteria having a large relative abundance (Supplementary Table S7). Overall, the *coxAB* gene was much higher than the *ccoNO* genes at all chimneys except Pagoda, which had the lowest relative abundance. There were large relative abundances of *coxAB* genes assigned in Alpha and Gammaproteobacteria in all the chimneys, and the Pagoda and Ultra-No-Chi-Chi Chimneys had a large relative abundance of Campylobacteria assigned to *coxAB* genes (Supplementary Tables S3–S7).

## 4 Discussion

The analysis of the metagenomes of five hydrothermal vent chimneys around the Pacific Ocean elucidates the differences in community composition and metabolic potential of chimneys of varying depth, age, and location. After examining the metagenomic contigs, the SSU rRNA taxonomic community compositions, and common genes involved in carbon fixation, nitrogen, sulfur, iron, arsenic, and oxygen metabolism in each chimney, it was found that each chimney had a distinct metabolic profile.

PERMANOVA showed no statistical significance to the chimneys clustering based on geographic location, activity, depth, or temperature; however, this is likely due to having no sample replicates. Despite a lack of statistically significant data, each chimney site has a unique community of microbes; however, there were noticeable trends. Similarities between the Snap-Snap and Ultra-No-Chi-Chi Chimneys are likely due to them being close in proximity and with similar chemical profiles. Similarities in metabolic potential and high prevalence of Gammaproteobacteria between the Castle Chimney and Ochre Chimney likely are due to the shallow depth of the Castle Chimney and the inactivity of the Ochre Chimney allowing for the growth of more aerobic microorganisms. This is supported by the low relative abundance of the *ccoNO*, indicating aerobic respiration is occurring in the presence of a high concentration of oxygen (Zhou et al., 2013).

The relative abundance of transposase genes in all five chimneys indicates horizontal gene transfer as a likely method of adaptation to the extreme environment of the chimneys (Reznikoff, 2003; Brazelton and Baross, 2009). Horizontal gene transfer among microbial taxa increases the phenotypic diversity of the chimney communities for microbes to better respond and adapt to environmental gradients (Brazelton and Baross, 2009). This is evident when examining extremophiles that commonly reside at hydrothermal vents. The extremophile *Fervidobacterium* showed transposases as indicators of horizontal gene transfer are common in thermophilic microbes (Cuecas et al., 2017).

The presence of an ammonia transport gene supports the high instance of ammonia oxidation at each of these chimneys. In addition, the ubiquity of ammonia transport genes across the chimneys suggests that microbes are accessing environmental nitrogen for assimilative or dissimilative processes (Sylvan et al., 2017).

Arsenic is inferred to be present at all five chimneys based on the ubiquity of the *arsC* gene. A diverse number of microbes can metabolize arsenic using *arsC* (Meyer-Dombard et al., 2013). Since nine different classes of microorganisms at these chimneys have an arsenic reductase gene, arsenic is likely present and detoxification is necessary for survival.

### 4.1 Ochre Chimney

The Ochre Chimney is an inactive, weathered chimney; therefore, the microbial community must gain its energy from the metabolism of solid minerals rather than the reduced chemicals present in the venting fluid of an active chimney. The Ochre chimney was dominated by Gammaproteobacteria as determined by amplicon sequencing, and many of the investigated genes were also identified as Gammaproteobacteria. The lack of venting fluid at inactive chimneys allows for more stable metabolic activity and cooler temperatures (Pan et al., 2022). At the Ochre Chimney, this is supported by the lower abundance of type I restriction enzymes and genes for chemotaxis. The lower relative abundance of these genes suggests less demand for microbes to respond quickly to changing chemical gradients since the sources of energy in the chimney sediment are relatively stable (Xie et al., 2011).

At the Ochre Chimney, carbon cycling at the inactive chimney is likely done via the CBB cycle since *rbcLS* has higher abundances than the other examined carbon cycling genes (Meier et al., 2019). Other studies examining the metabolic potential of inactive hydrothermal vent chimneys on the East Pacific Rise have identified the CBB cycle in autotrophic Gammaproteobacteria (Hou et al., 2020). In a study quantifying carbon fixation at inactive chimneys, it was found that Gammaproteobacterial CBB cycle genes were more prevalent than rTCA and Wood–Ljungdahl pathways (Achberger et al., 2024). Notably, Ochre also has two archaeal classes assigned to *rbcLS:* one in the Thaumarchaeota phylum and one at the unclassified Archaeal level. Archaeal RuBisCO genes are putatively involved in carbon dioxide fixation or AMP and nucleotide scavenging pathways (Beam et al., 2014). Thaumarchaeota, specifically Nitrososphaeria, are known to be common in inactive chimneys and can metabolize low concentrations of nitrogen and carbon (Han et al., 2018).

As *nirK* is largely used in archaea for ammonia oxidation and in bacteria for denitrification of nitrite, the relatively high number of different taxa that have a *nirK* gene for denitrification indicates an abundance of nitrite as an electron donor for lithotrophic growth (Kerou et al., 2016). At the hydrothermal vents of Explorer Ridge, nitrate was more prevalent than nitrite, which explained the high relative abundance of *nirK* (Tunnicliffe et al., 1986). The relatively large abundance of *amoA* genes could be due to increased ammonium found at the inactive chimney due to the breakdown of organic matter (Li J. et al., 2015). Both bacterial and archaeal *amoA* were identified and are likely critical in the nitrification process at the Ochre Chimney.

The availability of sulfur is a distinguishing factor in community composition between the inactive and active chimneys (Han et al., 2018). Since there are limited data on the hydrogen sulfide concentrations at the Ochre Chimney that come from previous analyses on nearby chimneys, the presence of different sulfur compounds can be inferred by the differential abundance genes for sulfur metabolism (Fullerton et al., 2024). Sulfate reduction by Deltaproteobacteria dominates at inactive chimneys, which in turn can change the mineral composition of the chimney with the production of pyrite (Han et al., 2018). The Gammaproteobacterial clade SUP05 has been shown to store sulfur, which allows it to metabolize sulfur when it may not be available from the chimney or its vent effluent (Shah et al., 2019). The co-occurrence of *dsrAB* and *soxAB* in certain taxa indicate that if dissimilatory sulfate reduction is occurring, thiosulfate oxidation could be occurring concurrently in the same taxa. This type of functional redundancy has been shown to increase ecological stability and resilience to disturbance, like the inactivation of a chimney (Biggs et al., 2020).

The relatively low abundance of Gammaproteobacterial *cyc2* genes at the Ochre Chimney indicates that iron oxidation may not be as prevalent at inactive, weathered chimney structures. Gammaproteobacteria are primary colonizers of inactive chimneys as they can oxidize sulfur present in the chimney structure (Hou et al., 2020). These Gammaproteobacteria may act as a catalyst in the weathering of inactive iron-sulfide chimneys, which could indicate that the Ochre Chimney was toward the end of the weathering process (Meier et al., 2019).

The *ccoNO* gene encodes a cbb_3_-type cytochrome c oxidase subunit I/II, which has a high affinity for oxygen and is more prevalent in lower oxygen concentrations, while the *coxAB* gene encodes an aa_3_-type cytochrome c oxidase subunit I/II, which has a low affinity for oxygen and is more prevalent in higher oxygen concentrations (Gier et al., 1996). The Ochre Chimney has a very small relative abundance of *ccoNO* cytochrome c oxidase genes compared to the other chimneys, further supporting that oxygen concentration is higher at this chimney.

### 4.2 Castle Chimney

The main pathway for carbon fixation at the Castle Chimney is seemingly via the CBB cycle, with most *rbcLS* genes associated with different Proteobacterial classes. Axial Seamount plumes and microbial mats have shown *Aquificae*, *Gammaproteobacteria*, *Campylobacteria*, and classes of methanogenic archaea dominated these microbial communities. Through metagenomics of another Axial Seamount chimney, Gammaproteobacteria were found to be the largest contributing taxon to the CBB cycle (Fortunato et al., 2018). Gammaproteobacteria tend to favor environments with higher oxygen and lower concentrations of sulfide (Meier et al., 2017). The higher concentration of oxygen present at the Castle Chimney may account for the high relative abundance of Gammaproteobacterial *rbcL* genes. The presumed high concentration of oxygen is also evidenced by the relatively low abundance of *ccoNO* genes. The rTCA cycle is favored over CBB in oxygen-limited environments, which could explain the higher relative abundance of Gammaproteobacterial CBB cycling genes over Campylobacterial rTCA cycling genes (Oulas et al., 2016). Another possible explanation is that these organisms are highly adapted to different environments due to sulfur, iron, or nitrogen cycling at each location, as evidenced by the Gammaproteobacterial clade SUP05’s ability to niche partition, store sulfur, and enhance carbon utilization with the use of thiosulfate (Marshall and Morris, 2013; Shah et al., 2019; Dede et al., 2022). For example, niche partitioning of both Gammaproteobacteria and Campylobacteria based on the concentration of sulfide as well as temperature has been previously demonstrated (Meier et al., 2017) and confirmed with the results of this analysis. The dominance of denitrification as evidenced by the large relative proportion of bacterial and archaeal *nirK* genes compared to the other chimneys is supported by previous analyses, which classified *nirK* transcripts to Thaumarchaeota and other ammonia-oxidizing archaea at Axial Seamount (Fortunato et al., 2018). Organisms that had the ammonia oxidation gene, *amoA*, always had *nirK* genes as well, indicating that these pathways could be co-occurring in ammonia-oxidizing archaea. The co-occurrence of metabolic pathways like these is hypothesized to be due to the decentralization of gene expression to maintain genetic diversity in variable environments such as hydrothermal vent chimneys (Carini et al., 2018).

The Castle Chimney likely has anaerobic sulfide-oxidizing bacteria since nitrite reduction genes and sulfur oxidation genes were identified as Gammaproteobacteria. As with Gammaproteobacterial sulfide oxidation via *dsrAB*, some Alphaproteobacteria couple denitrification via *nirK* with thiosulfate oxidation via *soxAB* in deep subsurface environments (Bell et al., 2020). Since many of these metabolic pathways have been shown to co-occur, it demonstrates that the microbes present in the Castle Chimney likely may be capable of gaining electrons from several different sources, as evidenced by the co-occurrence of Alphaproteobacterial *nirK* and *soxAB.*

The Castle Chimney iron oxidation is dominated by Gammaproteobacteria, a class that has been previously identified on the Juan de Fuca Ridge (Edwards et al., 2003). The Gammaproteobacterial clade SUP05 will partition niches based on the availability of iron and sulfide (Dede et al., 2022). Based on the high relative abundance of Gammaproteobacterial *cyc2, dsrAB*, and *soxAB*, the Castle Chimney likely has a large community of SUP05. Zetaproteobacteria, an iron-oxidizing bacteria commonly found at hydrothermal vents, are notably absent in the Castle Chimney. This could be due to the higher temperature and lower abundance of iron at Castle influencing a higher proportion of Gammaproteobacterial iron oxidation as Zetaproteobacteria tend to prefer lower temperatures (Mori et al., 2017).

### 4.3 Pagoda Chimney

Shaped like a mushroom or a Pagoda topped with a domed cap and many flanges coming out of the trunk, the Pagoda Chimney’s vent fluid is channelized through the flanges and up and over its cap, collecting in the center and creating several microenvironments of differing temperatures and chemistries (Teske et al., 2016). These different habitats introduce a need for microbes to adapt quickly to an ever-changing environment which is supported by the enrichment of transposase genes and therefore an increased potential for horizontal gene transfer (He et al., 2013). The microenvironments in Pagoda also allow for many different classes of microbes to reside, with the chimney dominated by Bacteroidetes, Campylobacteria, and Deltaproteobacteria. The Pagoda Chimney has the most taxonomic diversity of *rbcLS* genes, with a higher presence of archaeal *rbcLS* genes, likely due to the physical structure of the chimney allowing for many temperatures and chemical gradients (Böhnke and Perner, 2019). It has been shown that RuBisCO can also be used for nucleotide salvage rather than carbon fixation in archaea, which could explain the high abundance of archaeal *rbcLS* genes present (Wrighton et al., 2016).

Guaymas Basin is characterized by high phytoplankton productivity enhancing organic-rich sedimentation, thereby supporting hydrothermal vent fluid and sediment heterotrophic metabolisms (Teske et al., 2002). These organic-rich sediments lead to a large amount of hydrogen to be used for energy by organisms such as Methanopyri (Dombrowski et al., 2018). Sulfate reduction is common among microbial communities at Guaymas Basin hydrothermal chimneys. Previous metagenomic analysis of a chimney from the Guaymas Basin showed that heterotrophic sulfate-reducing bacteria were found at higher abundances due to the high concentrations of hydrocarbons (He et al., 2015). Sulfate-reducing bacteria degrade these plentiful hydrocarbons found at this site which in turn create H_2_ that can be used by methanogens in low-oxygen environments (He et al., 2013). Both the oxidative and reductive versions of *dsrAB* were present in Pagoda. The only instance of archaeal *dsrAB* genes present was in the Archaeoglobi class, the only known archaeal class that is hyperthermophilic with a sulfate-reducing metabolism (Pillot et al., 2021). As expected, Methanopyri was abundant in the Pagoda Chimney and likely utilizes the Wood–Ljungdahl pathway for carbon fixation and methanogenesis (Hügler and Sievert, 2011). Multiple hydrogenase uptake genes were found present at Pagoda, to further support *Methanopyri* methanogenesis. There is likely deoxygenation occurring inside the Pagoda chimney as supported by a low abundance of *coxAB* and *ccoNO* (Teske et al., 2016). This deoxygenation creates environments favorable for anaerobes such as *Methanopyri*.

### 4.4 Snap-Snap Chimney

Back-arcs, such as the Urashima Vent Field, can have a wide variation in pH, dissolved gasses, and metal concentrations due to variations in magma chemistry (Trembath-Reichert et al., 2019). The high abundances of genes for chemotaxis indicate a steep gradient of reduced chemicals needed for growth (Sourjik and Wingreen, 2012). The low-oxygen concentration is evidenced at Snap-Snap by the large relative abundance of *ccoNO* cytochrome c oxidase genes. Campylobacteria, Deltaproteobacteria, and Zetaproteobacteria were found at Snap-Snap both in the functional genes found through metagenomic sequencing and amplicon sequencing.

The Snap-Snap Chimney had a high abundance of Gammaproteobacterial *aclB* and *rbcLS* genes and the most *cbbM* genes out of all the chimneys, consistent with previous analyses of the CBB cycle on the Mariana back-arc (Trembath-Reichert et al., 2019). The presence of the *rbcLS* gene mapped to *Deinococci*, an extremophile chemoorganotroph. The high abundance of chemotaxis proteins could further indicate that the Snap-Snap Chimney is an extreme environment with variable chemical, temperature, and nutrient gradients.

Based on the relative abundances of dissimilatory nitrogen metabolism genes found at the Snap-Snap Chimney, a large amount of nitrite is likely used as an energy source (Levy-Booth et al., 2014). Previous analyses of archaeal denitrifiers have found that accumulation of organic material can increase *nirK* gene abundances, indicating that there may be organic material build-up at the Snap-Snap Chimney as there are archaeal *nirK* genes present (Hou et al., 2013).

At the Snap-Snap Chimney, there is a higher relative abundance of Campylobacteria than found at the other chimneys, indicating a high prevalence of reduced sulfur compounds in the vent fluid (Trembath-Reichert et al., 2019; Zhou et al., 2022). Deltaproteobacteria and Gammaproteobacteria have been found to have the ability to couple sulfate reduction with sulfur oxidation using the oxidative form of *dsrAB* (Müller et al., 2015). Since these taxa have both sulfur genes, coupled sulfur oxidation with sulfate reduction could be occurring.

Venting fluid at the Snap-Snap Chimney has been characterized by high concentrations of iron (Trembath-Reichert et al., 2019), which is hypothesized to be due to low pH from magmatic volatiles on the Mariana back-arc (Toki et al., 2015). The Snap-Snap Chimney has several taxa with the *cyc2* gene; however, no Zetaproteobacterial *cyc2* genes were identified in contrast to previous research (McAllister et al., 2020). This could be because it is a relatively new class with few isolated genomes. Therefore, the Zetaproteobacterial *cyc2* ORFs could be misidentified as Gammaproteobacterial or placed in an unclassified bacterial category during annotation (Emerson et al., 2007; Koeksoy et al., 2021).

### 4.5 Ultra-No-Chi-Chi Chimney

The Ultra-No-Chi-Chi Chimney was dominated by Campylobacteria, Alphaproteobacteria, and Zetaproteobacteria as identified in the genes present and by amplicon sequencing. The metabolisms of organisms at Ultra-No-Chi-Chi were relatively even when compared to the other chimneys. Notably, Ultra-No-Chi-Chi has nearly the same number of *aclB* and *rbcLS* genes, demonstrating that both the rTCA and CBB pathways are likely used for carbon fixation. The rTCA cycle is likely performed by Campylobacteria, while the CBB cycle has a higher diversity of taxa (Supplementary Table S7). The high relative abundance of both *ccoNO* and *coxAB* genes indicates that Ultra-No-Chi-Chi has a broad oxygen gradient, allowing for anaerobic and aerobic organisms to fix carbon.

Unexpectedly, Ultra-No-Chi-Chi has a lower abundance of genes coding for iron receptors and *cyc2*, which could indicate that there is less iron present. This was unexpected as other Urashima Chimneys have been characterized as iron-dominated (McAllister et al., 2020). However, Zetaproteobacterial *nirK* and SSU genes were found. Since iron oxidation is coupled with denitrification via *nirK* in Zetaproteobacteria (McAllister et al., 2021), Zetaproteobacterial iron oxidation is likely occurring at this chimney despite no *cyc2* genes mapping to that class.

## 5 Conclusion

Metagenomic analysis of these five hydrothermal vent chimneys demonstrates how the chemical composition of the chimney impacts the microbes that reside there and their potential metabolisms. Despite a unique collection of microorganisms, there were several uniting characteristics. Genes for DNA repair, chemotaxis, and transposases were present at higher abundances at hydrothermal vent chimneys compared to other environmental microbial communities, including terrestrial and marine soils, geothermal hot springs, the deep subsurface, the marine water column, and lake and estuary sediments and could be a uniting identifier for these communities to adapt to the ever-changing chemical and physical conditions. By both metagenomic and amplicon analyses, Gammaproteobacteria dominated the Ochre and Castle Chimneys while Campylobacteria were more prevalent at the Pagoda, Snap-Snap, and Ultra-No-Chi-Chi Chimneys. The relative abundances of oxygen and carbon metabolism genes at each of the chimneys tell a distinct story of the availability of these compounds as energy sources in both the active and inactive chimneys. A high relative abundance of oxygen metabolism genes coupled with the low relative abundance of carbon fixation genes could be used as a unique identifier for inactive chimneys, as shown with the Ochre Chimney. The differences in carbon fixation genes of the chimney microbes and the ability to shift their metabolic functional potential and community composition demonstrate that these microorganisms can adapt to varying chemical compositions of the chimneys and that many of these metabolic pathways tend to be functionally redundant to thrive in a dynamic ecosystem.

## Data availability statement

The amplicon and shotgun sequencing data sets generated for this study have been deposited in the NCBI Sequence Read Archive under the BioProject accession no. PRJNA996601.

## Author contributions

LM: Conceptualization, Data curation, Formal analysis, Investigation, Methodology, Validation, Writing – original draft, Writing – review & editing. HF: Conceptualization, Data curation, Formal analysis, Investigation, Methodology, Project administration, Resources, Software, Supervision, Validation, Visualization, Writing – original draft, Writing – review & editing. CM: Conceptualization, Data curation, Formal analysis, Funding acquisition, Investigation, Methodology, Project administration, Resources, Software, Supervision, Validation, Visualization, Writing – original draft, Writing – review & editing.
