# Supplementary material for: Microbial metabolic potential of hydrothermal vent chimneys along the submarine ring of fire
Source: Front Microbiol. 2024 Aug 6;15:1399422. doi: 10.3389/fmicb.2024.1399422 (PMC11333457; doi:10.3389/fmicb.2024.1399422)
Supplement: Supplementary file 1 [file Data_Sheet_1.PDF]

Supplemental Table 1. MAGs and their taxonomic assignments with completeness and contamination.

Bin	Taxa	Length	GC %	Number of Contigs	Disparity	Completeness	Contamination	Strain Heterogeneity
metabat2.48	Proteobacteria	1298003	56.2	248	0	52.49	0.84	100
metabat2.10	Deltaproteobacteria	1464749	47.93	255	0.032	50.1	0.8	0
metabat2.24	Bacteroidetes	2002372	45.87	404	0.036	43.27	0	0
metabat2.38	Actinobacteria	821088	64.03	239	0	41.31	4.76	50
metabat2.42	Bacteria	443507	40.84	77	0	41.14	3.61	0
metabat2.15	Calditrichaeota	782889	46.05	175	0.182	37.46	0	0
metabat2.63	Gemmatimonadetes	795028	62.62	180	0.124	34.56	0	0
metabat2.22	Deltaproteobacteria	1157082	47.48	63	0	34.19	0.07	0
metabat2.46	Spirochaetes	1267710	56.92	194	0.062	33.52	1.57	0
metabat2.40	Bacteria	543747	50.47	51	0	32.13	1.72	0
metabat2.2	Campylobacteria	688607	32.15	153	0.18	29.53	1.88	15.38
metabat2.26	Bacteria	1138543	50.56	133	0	27.74	1.72	0
metabat2.43	Bacteria	353357	37.65	72	0	26.8	1.88	0
metabat2.28	Bacteria	845593	50.56	179	0	26.02	0	0
metabat2.44	Bacteria	664143	46.57	85	0	24.14	0	0
metabat2.34	Actinobacteria	575531	69.38	168	0.037	22.91	0	0
metabat2.23	Bacteroidetes	432648	38.3	69	0	20.51	0	0
metabat2.45	Gemmatimonadetes	508301	49.59	80	0	18.97	0	0
metabat2.55	Deltaproteobacteria	573947	50.01	30	0	18.21	0	0
metabat2.56	Deltaproteobacteria	420040	47.14	70	0	17	0	0
metabat2.21	Bacteroidia	562460	41.13	128	0.099	16.89	0	0
metabat2.62	Gammaproteobacteria	441353	44.93	88	0	16.45	0.21	0

metabat2.27	Bacteroidetes	367914	39.52	44	0	16.41	0	0
metabat2.11	Campylobacteria	345372	34.05	97	0.152	16.24	0.2	40
metabat2.13	Bacteroidetes	582376	45.96	66	0	16.15	0	0
metabat2.17	Bacteroidetes	581793	39.05	76	0	16.07	0	0
metabat2.6	Flavobacteriia	444509	28.99	136	0.022	15.65	0.65	50
metabat2.20	Bacteria	482624	47.68	41	0	15.52	0	0
metabat2.54	Campylobacteria	355921	33.72	102	0.061	15.29	0.28	33.33
metabat2.51	Campylobacteria	277457	30.77	76	0	14.65	0	0
metabat2.58	Thermoplasmata	249099	44.04	25	0	14.21	0.28	0
metabat2.47	Campylobacteria	359569	30.37	112	0	13.93	1.28	0
metabat2.39	Calditrichaeota	588168	47.43	115	0.259	13.79	0	0
metabat2.32	Zetaproteobacteria	315076	52.37	56	0.049	13.22	0.09	0
metabat2.3	Gammaproteobacteria	290448	57.08	80	0	12.97	0	0
metabat2.33	Candidatus Gracilibacteria	232636	21.77	55	0.125	12.07	0	0
metabat2.16	Bacteria	220201	39.39	11	0	12.07	0	0
metabat2.36	Candidatus Cloacimonetes	205900	38.95	57	0.443	12.07	0	0
metabat2.60	Campylobacteria	307591	33.39	53	0.156	11.57	0.41	0
metabat2.61	Nitrospirae	243267	59.55	58	0.107	11.19	0	0
metabat2.31	Candidatus Gracilibacteria	242523	23.3	45	0.148	10.92	0.86	100
metabat2.41	Candidatus Bipolaricaulota	225858	66.05	41	0	10.85	0	0
metabat2.49	Nitrospinia	269898	53.43	55	0	10.34	0	0
metabat2.9	Bacteroidetes	224133	40.24	28	0	9.74	0.51	100
metabat2.29	Bacteroidetes	264380	44.2	39	0	9.56	0	0
metabat2.52	Bacteroidia	322320	40.13	83	0	9.27	0	0
metabat2.57	Campylobacteria	235128	32.98	49	0	9.23	0	0

metabat2.35	Betaproteobacteria	200733	44.25	34	0	9.22	0	0
metabat2.12	Candidatus Fermentibacteria	295485	49.17	55	0	8.62	0	0
metabat2.7	Alphaproteobacteria	288804	56.93	61	0	8.5	0	0
metabat2.19	Flavobacteriia	247701	30.52	67	0	8.2	0	0
metabat2.18	Bacteroidetes	211544	51.81	37	0	7.69	0	0
metabat2.8	Acidobacteria	297429	70.72	70	0.042	7.61	0	0
metabat2.64	Alphaproteobacteria	286760	54.95	60	0	7.43	0	0
metabat2.30	Gammaproteobacteria	258601	46.27	46	0.133	7.3	0	0
metabat2.1	Deltaproteobacteria	207298	45.56	27	0	7.2	0	0
metabat2.25	Bacteria	373313	48.42	87	0	6.11	0	0
metabat2.50	Deltaproteobacteria	606366	70.97	155	0.026	6	0	0
metabat2.5	Gammaproteobacteria	200928	48.79	44	0	5.39	0	0
metabat2.14	Bacteria	216750	47.92	24	0	5.17	0	0
metabat2.4	Campylobacteria	210151	34.45	34	0.305	4.93	0.16	0
metabat2.37	Acidimicrobiia	227805	62.45	53	0.233	2.52	0	0
metabat2.53	Gemmatimonadetes	217649	63.37	50	0.199	0	0	0

Supplemental Table 2. The Simpson and Shannon alpha diversity indices for the ASVs and Contigs from each Chimney.

Chimney	ASVs Simpson	Contig Simpson	ASVs Shannon	Contig Shannon
Ochre	0.997	0.750	6.928	2.161
Castle	0.954	0.830	4.428	2.131
Pagoda	0.989	0.890	5.466	2.818
Snap	0.976	0.800	4.997	2.143
Ultra	0.937	0.860	4.194	2.510

Supplemental Table 3. Relative abundance of different autotrophy genes and their taxonomic assignment for Ochre Chimney.

Taxa	*rbcS* (%)	*rbcL* (%)	*aclB* (%)	*cooS* (%)	*amoA* (%)	*nifH* (%)	*nirK* (%)	*dsrAB* (%)	*soxAB* (%)	*cyc2* (%)	*arsC* (%)	*ccoNO* (%)	*coxAB* (%)
Acidimicrobiia							4.72E-05						4.27E-03
Acidobacteria		3.22E-05					1.89E-04				4.29E-06		1.64E-02
Acidobacteriia													7.70E-05
Actinobacteria							1.07E-04				4.51E-05		1.50E-02
Actinomycetia		8.59E-06					2.58E-05				4.29E-06		1.73E-03
Alphaproteobacteria		2.06E-04				2.15E-06	9.45E-04	5.15E-05	2.77E-03	1.72E-05	1.27E-04		1.17E-01
Anaerolineae							3.65E-04						8.66E-04
Archaea		1.29E-05			7.24E-04		4.55E-03						2.19E-02
Ardenticatenia													3.85E-05
Bacilli											2.15E-06		
Bacteria	1.37E-03		4.29E-06	1.07E-05	2.15E-06		4.08E-03	6.66E-05	5.15E-05		2.23E-04	2.50E-04	9.21E-02
Bacteroidetes							1.07E-04			6.29E-04	8.59E-06	3.85E-05	1.62E-03
Betaproteobacteria		3.33E-04			2.88E-04		4.21E-04		1.72E-05		4.51E-05		1.10E-02
Calditrichae													6.73E-04
Calditrichaeota													1.35E-04
Campylobacteria			4.29E-06										1.92E-05
Candidatus Bipolaricaulota													3.85E-05
Candidatus Dadabacteria							1.07E-05				4.29E-06		1.54E-04
Candidatus Eisenbacteria													3.85E-05
Candidatus Omnitrophica							1.29E-05				1.29E-05		4.43E-04
Candidatus Peregrinibacteria													3.85E-05
Candidatus Tectomicrobia													1.35E-04
Chitinophagia													3.08E-04
Chlorobi												3.85E-05	
Chloroflexi		2.15E-05					3.80E-04				3.86E-05	1.15E-04	6.15E-02

Crenarchaeota							4.29E-06						
Cyanobacteria		2.00E-04											
Cytophagia							4.29E-06					1.35E-04	2.69E-04
Dehalococcoidia													9.62E-04
Deinococci													3.85E-05
Deltaproteobacteria		3.44E-05		4.29E-06	8.59E-06		2.15E-04	4.29E-06		4.66E-05	3.01E-05	7.70E-05	1.71E-02
Elusimicrobia							4.29E-06						2.31E-04
Flavobacteriia							9.02E-05					1.92E-04	2.12E-04
Gammaproteobacteria		4.72E-04			1.50E-05		9.45E-04	1.72E-03	2.25E-03	1.04E-02	1.86E-03		1.19E-01
Gemmatimonadetes		1.22E-04					3.56E-04				7.51E-05	9.04E-04	2.88E-02
Ignavibacteria											4.29E-06		1.10E-03
Nitrososphaeria					7.06E-04		1.08E-02				6.44E-05		5.37E-02
Nitrospinae							1.37E-04		1.29E-05		1.10E-04		1.58E-03
Nitrospinia							1.85E-04				5.37E-05	7.70E-05	7.70E-05
Nitrospira			3.44E-05				4.29E-05						6.93E-04
Nitrospirae		1.29E-05	2.45E-04				1.57E-04				9.45E-05		9.24E-04
Oligoflexia													3.85E-05
Opitutae													1.15E-04
Phycisphaerae													9.24E-04
Planctomycetes							1.07E-05					1.35E-04	6.52E-03
Planctomycetia											4.29E-06	1.92E-05	1.31E-03
Proteobacteria		2.68E-03			8.59E-06		2.87E-03	7.88E-04	1.48E-03		3.78E-04	2.12E-04	6.67E-02
Rhodothermae		5.37E-05					3.22E-05						
Saprospiria													3.85E-05
Spirochaetia													3.85E-05
Thaumarchaeota		1.07E-04			1.47E-03		3.31E-03						3.84E-02
Verrucomicrobia							8.59E-06						3.46E-04
Verrucomicrobiae													1.15E-04
**TOTAL**	1.37E-03	4.19E-03	8.58E-06	1.07E-05	1.75E-03	2.15E-06	2.71E-02	1.84E-03	5.10E-03	6.93E-04	2.81E-03	1.96E-03	6.84E-01

Supplemental Table 4. Relative abundance of different autotrophy genes and their taxonomic assignment for Castle Chimney.

Taxa	*rbcS* (%)	*rbcL* (%)	*aclB* (%)	*cooS* (%)	*amoA* (%)	*nifH* (%)	*nirK* (%)	*dsrAB* (%)	*soxAB* (%)	*cyc2* (%)	*arsC* (%)	*ccoNO* (%)	*coxAB* (%)
Acidimicrobiia													4.24E-03
Acidithiobacillia		6.00E-04											
Acidobacteria		1.55E-05						1.03E-04					1.02E-03
Acidobacteriia													1.46E-04
Actinobacteria											9.71E-06		2.78E-03
Actinomycetia				5.83E-06									4.87E-05
Alphaproteobacteria		4.74E-04				3.30E-05	2.23E-04	1.23E-03	2.54E-03		4.08E-05		1.47E-02
Anaerolineae													2.92E-04
Ardenticatenia													1.53E-03
Bacteria	3.46E-03			3.89E-03		1.33E-03	7.19E-03	2.49E-04	1.03E-02		1.05E-04	1.39E-03	8.45E-02
Bacteroidetes							5.95E-04			6.29E-04	2.56E-04	1.13E-02	1.46E-04
Bacteroidia							6.80E-05						
Betaproteobacteria		3.44E-04			1.94E-05	1.55E-05	6.41E-05	4.45E-04	3.59E-04		6.02E-05		3.41E-04
Campylobacteria			7.77E-06						1.94E-06				4.87E-05
Candidatus Bipolaricaulota													4.87E-05
Candidatus Omnitrophica				1.94E-06									
Candidatus Tectomicrobia													2.44E-05
Chlorobia				3.89E-06									
Chloroflexi		2.78E-04											3.17E-03
Chloroflexia							1.94E-06						
Cyanobacteria		1.94E-05											
Cyanophyceae		1.94E-05				3.89E-06							
Cytophagia													4.87E-05
Deinococci													4.14E-04
Deltaproteobacteria				2.80E-04		2.56E-04	1.75E-05	2.68E-04	2.08E-04	4.66E-05	1.17E-05	1.22E-04	1.85E-03

Elusimicrobia		1.36E-05											
Euryarchaeota							1.17E-05						
Firmicutes						3.89E-06							
Flavobacteriia							3.69E-05				1.17E-05	1.46E-04	1.95E-04
Gammaproteobacteria		1.51E-02		1.40E-03	2.72E-05	5.07E-04	3.63E-04	2.46E-02	5.02E-03	1.04E-02	2.21E-03	4.12E-03	1.32E-01
Gemmatimonadetes		1.94E-06									1.75E-05		9.74E-05
Halobacteria							1.88E-04						
Holophagae													4.87E-05
Hydrogenophilalia		1.75E-05				1.94E-06							
Ignavibacteria										3.85E-04			
Methanopyri						1.94E-06							
Nitrososphaeria					7.77E-06		2.72E-05						4.63E-04
Nitrospinae							1.94E-06				2.72E-05		2.44E-05
Nitrospira			5.83E-06				3.89E-06						
Nitrospirae			3.11E-05	4.21E-03		1.24E-03		3.05E-03					6.94E-03
Planctomycetes				7.77E-06			1.55E-05					9.74E-05	1.32E-03
Planctomycetia											9.13E-05	9.74E-05	4.87E-04
Proteobacteria		1.08E-02		2.27E-04	7.77E-06	1.11E-02	3.42E-04	1.04E-02	2.86E-03	2.33E-05	3.42E-04		1.19E-01
Saprospiria													4.87E-05
Thaumarchaeota					7.77E-06		1.17E-05						
Verrucomicrobia												9.74E-05	
Zetaproteobacteria		1.94E-06											
**TOTAL**	3.46E-03	2.77E-02	4.47E-05		6.99E-05	1.45E-02	9.16E-03	4.03E-02	2.13E-02	1.15E-02	3.18E-03	1.73E-02	2.57E-01

Supplemental Table 5. Relative abundance of different autotrophy genes and their taxonomic assignment for Pagoda Chimney.

Taxa	*rbcS* (%)	*rbcL* (%)	*aclB* (%)	*cooS* (%)	*amoA* (%)	*nifH* (%)	*nirK* (%)	*dsrAB* (%)	*soxAB* (%)	*cyc2* (%)	*arsC* (%)	*ccoNO* (%)	*coxAB* (%)
Acidobacteria								1.48E-03			1.30E-04	5.21E-05	2.60E-05
Alphaproteobacteria		6.69E-06						1.34E-05	2.68E-05				4.17E-04
Anaerolineae											2.01E-05		5.94E-03
Aquificae			1.34E-05						1.00E-05				
Archaea		5.55E-04	1.07E-04	7.36E-05				5.02E-05					
Archaeoglobi		1.34E-05		1.34E-05				4.01E-05					
Armatimonadetes				3.01E-05									
Bacteria	1.46E-03		9.10E-04	1.20E-03		1.00E-05	1.08E-03	4.38E-03	1.94E-04	5.69E-05	6.76E-04	8.86E-03	2.56E-02
Bacteroidetes		6.69E-05					7.59E-04			6.69E-06	2.94E-03	4.26E-02	4.06E-03
Bacteroidia											1.34E-05	2.08E-03	2.60E-04
Betaproteobacteria													7.81E-05
Caldilineae		3.35E-06											2.34E-04
Calditrichae											5.35E-05		
Calditrichaeota											5.69E-05		
Campylobacteria			3.59E-03						1.03E-03		9.33E-04		4.71E-02
Candidatus Bipolaricaulota			1.96E-03						1.00E-05				
Candidatus Hydrogenedentes				3.35E-06									
Candidatus Korarchaeota				3.01E-05									
Candidatus Lokiarchaeota		6.68E-06											
Candidatus Peregrinibacteria		1.34E-05											
Candidatus Sumerlaeota											8.70E-05		
Candidatus Thermoplasmatota		6.02E-05											
Candidatus Woesarchaeota		3.01E-05											
Chitinophagia													1.56E-04
Chlorobi											3.35E-04	2.21E-03	1.59E-03
Chloroflexi		2.78E-04	9.03E-05	1.74E-03		1.67E-05	8.03E-05		6.69E-06				8.15E-03
Crenarchaeota		4.35E-05											

Cytophagia												2.84E-03	5.21E-05
Deinococci		3.35E-06											
Deltaproteobacteria		6.02E-05		3.75E-03		1.24E-04		1.58E-03	1.00E-05				1.67E-03
Euryarchaeota		1.34E-05		4.01E-05		1.00E-05							
Firmicutes				1.67E-05		1.67E-05					1.00E-05		
Flavobacteriia											1.07E-04	1.46E-03	
Gammaproteobacteria		5.35E-05		1.34E-05		3.35E-06		1.38E-03	2.68E-05	1.67E-05	1.10E-04		1.09E-03
Gemmatimonadetes								3.35E-06				2.34E-04	
Ignavibacteriae											4.35E-05	1.04E-04	
Methanomicrobia		4.35E-05		1.00E-05									
Methanopyri				4.62E-03		1.80E-03							
Nitrospinae													5.21E-05
Nitrospirae				6.69E-06		6.69E-06		6.69E-06					5.21E-05
Phycisphaerae													1.30E-04
Proteobacteria		3.58E-04		1.80E-03	1.00E-05	4.01E-05	6.69E-06	6.02E-05			1.47E-04		3.91E-04
Spirochaetes											4.01E-05		
Thermococci		8.10E-04		9.94E-04									
Thermodesulfobacteria				6.69E-06				2.01E-05					
Thermoplasmata			5.19E-04										
Thermoprotei		7.69E-05		9.37E-05									
Verrucomicrobia							4.35E-05					1.38E-03	1.12E-03
Verrucomicrobiae												4.17E-04	
**TOTAL**	1.46E-03	2.42E-03	6.67E-03	1.43E-02	1.00E-05	1.99E-03	1.93E-03	8.99E-03	1.29E-03	6.36E-05	5.52E-03	6.04E-02	9.71E-02

Supplemental Table 6. Relative abundance of different autotrophy genes and their taxonomic assignment for Snap-Snap Chimney.

Taxa	*rbcS* (%)	*rbcL* (%)	*aclB* (%)	*cooS* (%)	*amoA* (%)	*nifH* (%)	*nirK* (%)	*dsrAB* (%)	*soxAB* (%)	*cyc2* (%)	*arsC* (%)	*ccoNO* (%)	*coxAB* (%)
Acidimicrobiia											9.68E-06		1.26E-03
Acidithiobacillia		1.45E-05											
Acidobacteria								1.99E-04	4.84E-06		1.94E-05	2.64E-03	2.03E-03
Acidobacteriia													8.12E-05
Actinobacteria							2.66E-05				4.84E-06		8.12E-05
Actinomycetia													3.25E-04
Alphaproteobacteria		1.67E-03					1.62E-03	1.39E-03	2.79E-03	9.68E-06	6.17E-04		1.08E-01
Aquificae			2.42E-05						4.14E-04				7.63E-03
Archaea		3.63E-05	4.60E-05	1.45E-05									
Archaeoglobi		4.60E-05		9.68E-06		4.84E-06		3.87E-05					
Ardenticatenia							7.26E-06						
Bacteria	3.27E-04		4.84E-06	3.29E-04		6.05E-05	4.50E-04	6.46E-04	8.09E-04	9.76E-04	8.21E-04	2.76E-03	2.33E-02
Bacteroidetes							3.39E-04			7.19E-04	2.18E-04	2.48E-02	2.88E-03
Bacteroidia							1.91E-04		4.84E-06				
Betaproteobacteria		6.78E-05			4.84E-06		1.69E-05				2.18E-05		9.74E-04
Caldilineae													1.62E-04
Calditrichaeota									2.42E-06		9.68E-06		8.12E-05
Candidatus Bipolaricaulota									9.68E-06				
Candidatus Heimdallarchaeota		4.84E-06											8.12E-05
Candidatus Lambdaproteobacteria													2.43E-04
Candidatus Omnitrophica													8.12E-05
Candidatus Sumerlaeota											2.42E-05	4.06E-04	4.87E-04
Chitinophagia													4.06E-04
Chlorobi											9.68E-06	2.84E-03	8.93E-04
Chloroflexi		4.84E-06		1.45E-05								8.12E-05	2.84E-03
Cytophagia												3.25E-04	6.09E-04
Dehalococcoidia								4.84E-06					

Deinococci		3.14E-03					5.57E-05				9.93E-05		1.93E-02
Deltaproteobacteria				4.20E-03		1.12E-03	4.84E-06	4.15E-03	4.02E-04			8.12E-05	4.38E-03
Campylobacteria			2.36E-02						1.40E-02		1.11E-02		1.03E+00
Euryarchaeota		9.68E-06											8.12E-05
Flavobacteriia							7.75E-05				5.04E-04	3.11E-02	4.63E-02
Gammaproteobacteria		3.14E-03			3.39E-05		1.67E-04	3.45E-03	3.46E-03	2.64E-04	7.26E-04		1.14E-01
Ignavibacteriae											4.84E-06	8.12E-05	6.90E-04
Methanomicrobia		2.42E-06											
Nitrososphaeria							2.42E-05						7.30E-04
Nitrospinae													1.06E-03
Nitrospira							1.69E-05						
Nitrospirae			9.68E-06	1.33E-04				7.26E-05	7.75E-05				
Oligoflexia												8.12E-05	3.65E-04
Phycisphaerae												2.43E-04	5.68E-04
Planctomycetes											4.84E-06	9.74E-04	1.95E-03
Planctomycetia													2.35E-03
Proteobacteria		4.31E-03		2.66E-05		2.42E-06	2.16E-03	5.25E-04	1.34E-03		2.33E-03	3.65E-04	2.53E-02
Saprospiria													4.06E-05
Sphingobacteriia												8.12E-05	
Spirochaetia													8.12E-05
Thaumarchaeota							2.66E-05						1.62E-04
Thermococci		1.45E-05											
Thermodesulfobacteria				9.44E-05		2.18E-05		1.14E-04					
Thermoprotei		1.45E-05											
Thermotogae							4.84E-06						8.12E-05
Verrucomicrobia							1.45E-05				2.18E-05		3.25E-04
Verrucomicrobiae							4.84E-06						8.12E-05
Zetaproteobacteria		1.14E-03					2.32E-04				6.15E-04	6.49E-04	1.15E-02
**TOTAL**	3.27E-04	1.25E-02	2.37E-02	4.73E-03	4.84E-06	1.19E-03	5.19E-03	1.05E-02	2.20E-02	1.70E-03	1.65E-02	6.68E-02	1.40E+00

Supplemental Table 7. Relative abundance of different autotrophy genes and their taxonomic assignment for Ultra-No-Chi-Chi Chimney.

Taxa	*rbcS* (%)	*rbcL* (%)	*aclB* (%)	*cooS* (%)	*amoA* (%)	*nifH* (%)	*nirK* (%)	*dsrAB* (%)	*soxAB* (%)	*cyc2* (%)	*arsC* (%)	*ccoNO* (%)	*coxAB* (%)
Acidithiobacillia		2.36E-05											
Acidimicrobiia											5.23E-06		8.43E-05
Acidobacteria								4.58E-04	5.23E-06		4.45E-05	8.18E-03	7.55E-03
Acidobacteriia													8.43E-05
Actinobacteria													1.26E-04
Actinomycetia											2.62E-06		
Alphaproteobacteria		3.82E-03						4.10E-03	6.26E-03		1.42E-03		9.64E-02
Anaerolineae													4.34E-03
Aquificae			8.27E-04	9.92E-04					1.86E-03	1.31E-05			1.99E-02
Archaea		4.03E-04	5.23E-06	9.16E-05									7.17E-04
Archaeoglobi		6.52E-04		1.15E-04		1.02E-04		1.38E-03					3.37E-04
Armatimonadetes				2.62E-06									
Bacteria	1.54E-04		1.57E-04	6.67E-04		1.15E-04	2.38E-04	1.51E-03	1.89E-03		1.73E-03	3.38E-02	9.16E-02
Bacteroidetes							1.78E-04				3.64E-04	7.80E-03	1.55E-02
Betaproteobacteria		1.05E-04											
Caldilineae								1.05E-05					1.35E-03
Calditrichae	3.66E-05												9.70E-04
Calditrichaeota		3.40E-05									1.02E-04		9.28E-04
Candidatus Bathyarchaeota				3.66E-05									
Candidatus Bipolaricaulota									2.36E-05				
Candidatus Heimdallarchaeota		3.32E-04											3.25E-03
Candidatus Hydrogenedentes													4.22E-04
Candidatus Marinimicrobia													9.28E-04
Candidatus Microgenomates		5.23E-06											
Candidatus Pacearchaeota		1.57E-05											
Candidatus Sumerlaeota											5.76E-05	2.70E-03	1.22E-03
Chitinophagia													1.69E-04
Chlorobi											5.76E-05	1.45E-02	1.26E-02
Chloroflexi		1.05E-05		3.93E-05			1.62E-04					6.75E-04	3.60E-02

Cytophagia													8.43E-04
Deinococci		3.00E-04					1.31E-04				2.04E-04		5.00E-02
Deltaproteobacteria				1.68E-03		5.50E-05		1.50E-03	2.36E-05			1.48E-03	1.05E-02
Epsilonproteobacteria			1.97E-02						1.22E-02		1.22E-02		8.69E-01
Euryarchaeota		2.62E-05		3.14E-05				1.05E-05					2.40E-03
Flavobacteriia							2.43E-04				3.30E-04	2.00E-02	2.96E-02
Gammaproteobacteria		1.48E-03					8.64E-05	3.17E-03	5.85E-03		2.26E-03		1.32E-01
Halobacteria													4.22E-05
Ignavibacteriae											4.71E-05	8.01E-04	3.42E-03
Methanomicrobia				1.57E-05									
Nitrososphaeria													3.37E-04
Nitrospinae			1.05E-05						7.33E-05				4.22E-04
Nitrospirae				1.46E-03		3.66E-05		1.04E-03	5.99E-04				
Oligoflexia												8.01E-04	3.37E-04
Planctomycetes							3.40E-05					7.38E-03	7.17E-03
Planctomycetia													2.53E-04
Proteobacteria		1.06E-02		4.71E-05		2.62E-06	2.54E-04	7.24E-04	1.75E-04	8.64E-05		4.22E-04	1.82E-02
Saprospira		2.62E-06										4.22E-05	1.69E-04
Spirochaetes													3.37E-04
Spirochaetia							2.62E-05						1.94E-03
Thaumarchaeota													1.69E-04
Thermococci		4.19E-05		1.81E-04									
Thermodesulfobacteria				2.17E-04		2.62E-05		2.38E-04					
Thermomicrobia													1.26E-04
Thermoprotei		2.41E-04		2.62E-05									
Thermotogae							4.97E-05						5.14E-03
Verrucomicrobia												4.22E-05	
Zetaproteobacteria		3.64E-04					1.67E-04					1.26E-04	5.57E-03
**TOTAL**	1.91E-04	1.85E-02	2.07E-02	5.60E-03		3.37E-04	1.57E-03	1.41E-02	2.90E-02	9.95E-05	1.88E-02		1.43E+00

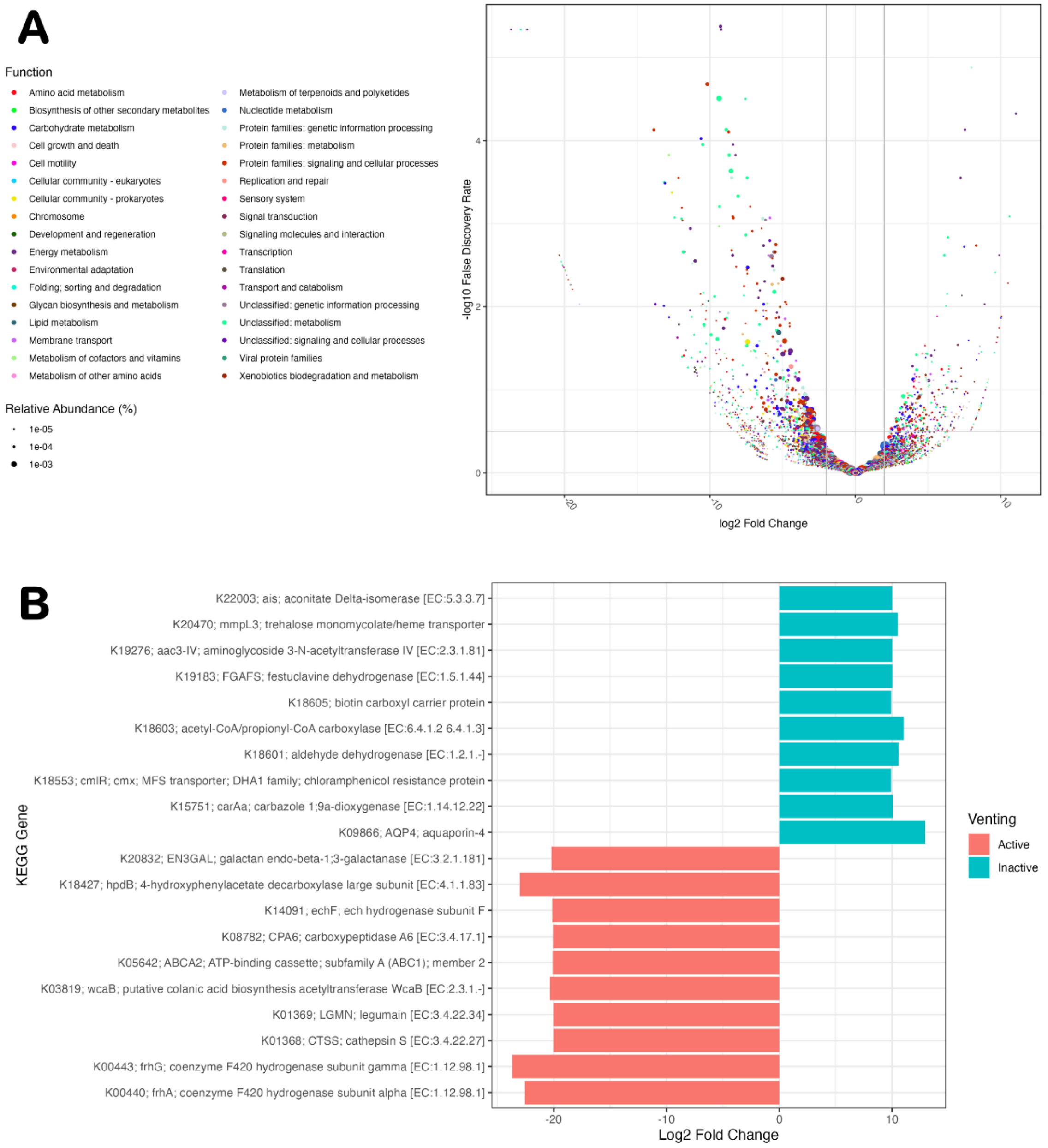

Supplemental Figure 1. (A) Volcano plot of differentially expressed genes. Color of each point indicates gene function group and size indicates the percent relative abundance of each gene. P-values were less than or equal to 0.05. (B) Top 10 most differentially expressed KEGG genes between active and inactive chimneys. P-values were less than or equal to 0.00037.

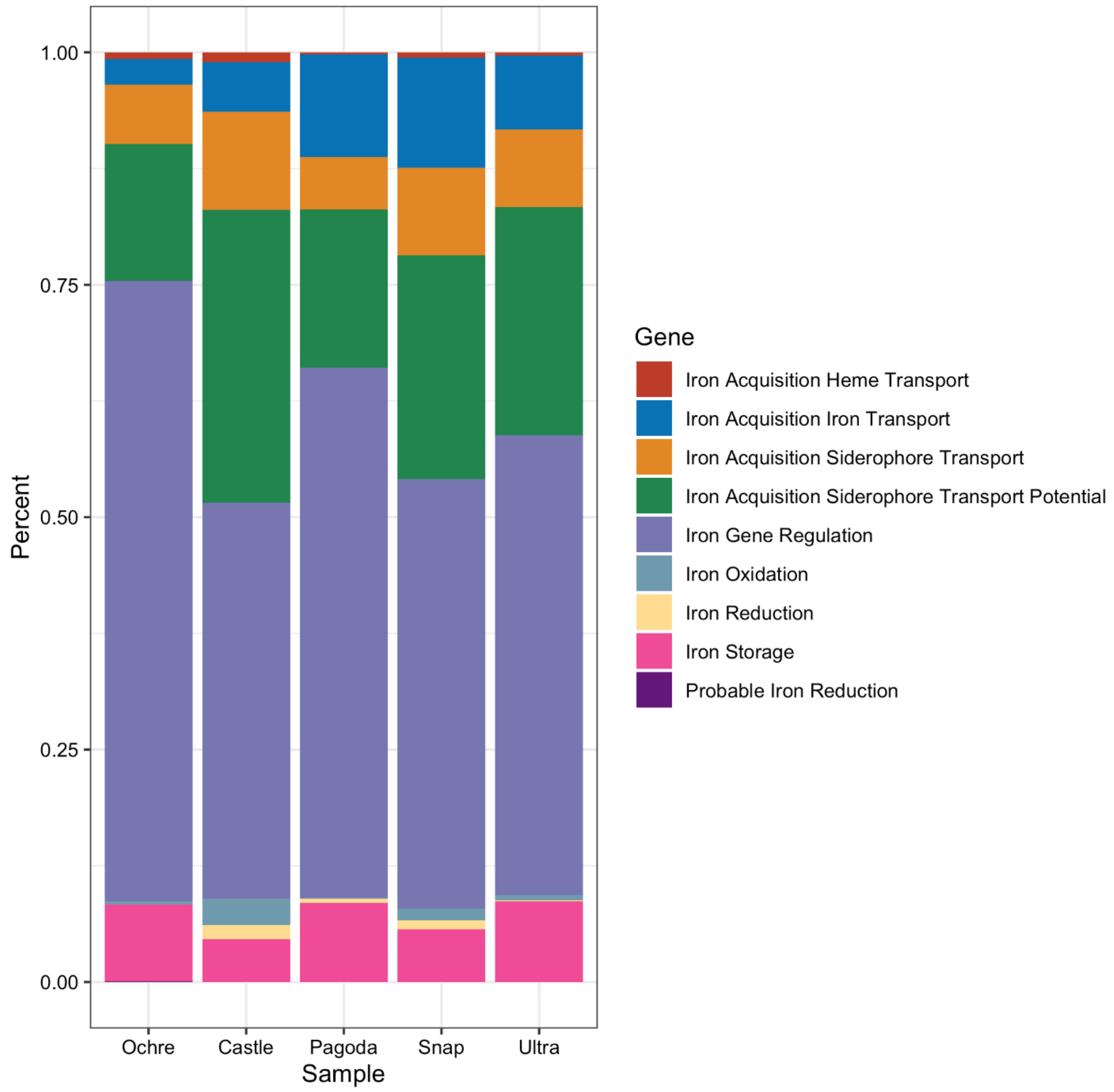

Supplemental Figure 2. Stacked bar graph as a percentage of the whole of different types of iron genes found in each chimney from the FeGenie analysis.
